# Propolis Exerts an Anti-Inflammatory Effect on PMA-Differentiated THP-1 Cells via Inhibition of Purine Nucleoside Phosphorylase

**DOI:** 10.3390/metabo9040075

**Published:** 2019-04-16

**Authors:** Abdulmalik M. Alqarni, Kanidta Niwasabutra, Muhamad Sahlan, Hugo Fearnley, James Fearnley, Valerie A. Ferro, David G. Watson

**Affiliations:** 1 Strathclyde Institute of Pharmacy and Biomedical Sciences, University of Strathclyde, 161 Cathedral Street, Glasgow G4 0RE, UK; kanidta.niwasabutra@strath.ac.uk (K.N.); v.a.ferro@strath.ac.uk (V.A.F.); d.g.watson@strath.ac.uk (D.G.W.); 2 Department of Pharmaceutical Chemistry, College of Clinical Pharmacy, Imam Abdulrahman Bin Faisal University (University of Dammam), Dammam 31441, Saudi Arabia; 3 Faculty of Engineering, Universitas Indonesia Campus UI, Depok 16424, Indonesia; muhamad.sahlan@gmail.com; 4 Apiceutical Research Centre, 6 Hunter Street, Whitby, North Yorkshire YO21 3DA, UK; hugofearnley@beearc.com (H.F.); james.fearnley@beearc.com (J.F.)

**Keywords:** propolis, pro- and anti-inflammatory cytokines, LPS stimulation, THP-1 cells, PMA differentiated, macrophages

## Abstract

Previous research has shown that propolis has immunomodulatory activity. Propolis extracts from different geographic origins were assessed for their anti-inflammatory activities by investigating their ability to alter the production of tumour necrosis factor-α (TNF-α) and the cytokines interleukin-1β (IL-1β), IL-6 and IL-10 in THP-1-derived macrophage cells co-stimulated with lipopolysaccharide (LPS). All the propolis extracts suppressed the TNF-α and IL-6 LPS-stimulated levels. Similar suppression effects were detected for IL-1β, but the release of this cytokine was synergised by propolis samples from Ghana and Indonesia when compared with LPS. Overall, the Cameroonian propolis extract (P-C) was the most active and this was evaluated for its effects on the metabolic profile of unstimulated macrophages or macrophages activated by LPS. The levels of 81 polar metabolites were identified by liquid chromatography (LC) coupled with mass spectrometry (MS) on a ZIC-pHILIC column. LPS altered the energy, amino acid and nucleotide metabolism in THP-1 cells, and interpretation of the metabolic pathways showed that P-C reversed some of the effects of LPS. Overall, the results showed that propolis extracts exert an anti-inflammatory effect by inhibition of pro-inflammatory cytokines and by metabolic reprogramming of LPS activity in macrophage cells, suggesting an immunomodulatory effect.

## 1. Introduction

In recent years, the discovery and development of new and existing anti-inflammatory therapies have been an intense research focus, particularly for the control of chronic inflammatory conditions. Inflammatory cells, such as leukocytes, mast cells, endothelial cells, monocytes, macrophages and lymphocytes, are now recognised to produce chemical inflammatory mediators that have the purpose of repairing tissue injury [1]. These mediators include amines (histamine and serotonin), arachidonic acid, eicosanoids, leukotrienes, prostaglandins, cytokines (tumour necrosis factor alpha, TNF-α and interleukins, IL) and free oxygen radicals [2,3]. The inflammatory process can be categorised into acute and chronic stages, according to the duration and frequency of the injurious agent. The acute-stage response includes supportive or exudative, as well as cellular and microvascular, actions. By contrast, chronic inflammation is proliferative and the resulting histological alterations differ from those in the acute stage to include cell migration and mitotic activity [4,5].

In certain instances, inflammation needs to be regulated by specific drugs to avoid further consequences to the organism. Current anti-inflammatory treatments, which include nuclear factor kappa B (NF-κB) inhibitors, anti-cytokine antibodies, anti-inflammatory cytokines, enzyme inhibitors and kinase inhibitors, can be classified according to their mechanisms of action. Modulation of different signal transduction pathways having similar endpoints (e.g., TNF-α inhibition) might induce different cellular reactions, thereby giving rise to the observed complexity of the inflammatory process [6]. Therefore, high throughput screening methods need to be applied in the drug discovery process.

Currently, several drug discovery studies have been targeted to investigate the direct health benefits and pharmacological properties of honey bee products. One of the most common is propolis, also known as bee glue, which is a resinous substance collected by bees from their surrounding environment (namely from plants) [7]. The wide range of biological activities of propolis is due to the presence of a complex mixture of bioactive compounds, which impart antioxidant, antimicrobial, anticancer and anti-inflammatory actions [8,9,10]. The effective medical applications of propolis have led to an increased interest in its chemical composition, which is highly variable depending on the climate and environmental conditions of the site, the collecting season and the geographic region [11]. Over 300 compounds have been identified in propolis, with the most abundant being phenolics (e.g., flavonoids, flavanones and flavonolols), aromatic aldehydes, steroids, alcohols, fatty acids, terpenes, amino acids and sugars [10,12,13]. The chemical compounds also differ between propolis samples originating from tropical and temperate zones. For example, the phenolic constituents in temperate zone propolis include the flavonoids pinocembrin, chrysin, ferulic acid, cinnamic acid and caffeic acid [14,15], whereas propolis from tropical areas contains only traces of these phenolic components, but is rich in prenylated derivatives of benzophenones, p-coumaric acid, lignans and diterpenes [16,17,18].

Many of these propolis components have anti-inflammatory action and can act on common and/or distinct anti-inflammatory pathways that function in basic immune cell responses. One example is the pathway that involves special receptors, the Toll-like receptors (TLRs), which recognise various microbial structures called pathogen-associated molecular patterns (PAMPs). Consequently, several transcriptional factors (e.g., NF-κB) are activated and lead to gene expression and the release of inflammatory cytokines to promote host defence [19]. This response is mediated by B and T cells and results in pathogen-specific adaptive immunity [20].

Previous studies have shown that propolis components have direct regulatory action on these basic immune cell functions. For instance, neovestitol, an isoflavonoid from propolis, had an immune modulatory effect in lipopolysaccharide (LPS)-stimulated RAW264.7 macrophages, where it clearly inhibited nitric oxide (NO) production and reduced pro-inflammatory cytokine levels [21]. In Th1- and Th2-type T cells, propolis extracts and propolis compounds (e.g., caffeic acid, phenethyl ester, quercetin and hesperidin) strongly depress DNA synthesis and the production of inflammatory cytokines (i.e., IL-1β, IL-12, IL-2 and IL-4) and enhance the production of transforming growth factor-β1 (TGF-β1) [22]. Large amounts of apigenin, galangin and pinocembrin were quantified by flavonoid profiling of propolis from southern Brazil [23]. Zhang et al. reported the ability of apigenin to decrease the mRNA levels of IL-1β, IL-6 and TNF-α in human THP-1-derived macrophages [24]. The levels of these pro-inflammatory cytokines were also significantly decreased by pinocembrin in RAW 264.7 macrophage cells, whereas IL-10 was significantly increased [25]. In the same RAW 264.7 line, the level of IL-6 and TNF-α cytokines was clearly reduced by galangin [26]. In vivo, propolis administration to C57BL/6 mice for 14 days led to inhibition in the production of IL-1β, IL-6, IL-2, IL-10 and IFN-γ by spleen cells [27]. In addition, ethanolic extract of Brazilian propolis reduced the expression of IL-17 in collagen-induced arthritis in mice [28]. Thus, bee propolis and its constituents can be considered as potential natural anti-inflammatory agents that act by modulating immune responses.

New compounds have recently been reported from propolis samples from Africa, including two new stilbene compounds isolated from Ghanaian samples and a new phloroglucinonone compound isolated from Cameroon samples [29]. Cameroonian propolis has been used in traditional medicine as an antibacterial and antiradical agent [30,31]. Chemical investigations by Kardar et al. of the triterpenes and phenolic compounds in Cameroonian propolis led to the isolation of 13 alk(en)ylphenols, nine triterpenes and nine alk(en)ylresorcinols [32,33]. This propolis also contained diprenyl flavonoids, two monoterpenic alcohols and one fatty acid ester, as reported previously [34]. Only a few reports have used Cameroonian propolis to study the anti-inflammatory effects of propolis; however, the assessment of its antagonist effects on LPS activation of macrophage cells could be improved by the use of a metabolomics approach.

Metabolomics is a recently introduced tool that has now joined genomics, transcriptomics and proteomics for the analysis of biological systems [35]. The use of metabolomics analysis for metabolic profiling has attracted increasing interest, as it allows for simultaneous and reproducible recognition of both endogenous and exogenous metabolites that could directly reflect the biological alterations in a test sample. Conventionally, a biomarker from a metabolomics study is achieved by comparing the metabolic profile between two states (e.g., control versus treatment or health versus disease) [36]. For research on inflammatory diseases, metabolomics has been highlighted as a promising analytical technique for identifying particular metabolites or metabolic pathways associated with a disease. For example, possible clinically useful metabolic biomarkers for patients with Crohn’s disease were identified as tyrosine and phenylalanine metabolism and bile acid and fatty acid biosynthesis, based on non-targeted metabolic profiling of faecal samples [37]. Similarly, comprehensive metabolic analysis of LPS-stimulated and unstimulated macrophage cells by LPS [38] following targeted metabolic profiling of individual pathways, such as amino acids [39], carbohydrates [40] and lipids [41], provided a broad determination of the pathogenic mechanism involved in inflammatory macrophage biology and/or disease.

Macrophages, upon stimulation with LPS, perform a multitude of functions for tissue remodelling and immune responses, and they secrete a wide range of factors associated with inflammatory pathways, including pro-inflammatory cytokines, growth factors and prostaglandins [42]. The effects on the characteristic metabolic changes that LPS-activated macrophages undergo could help to assess the activities of anti-inflammatory compounds. In the present study, we assessed propolis samples from different regions (UK, Ghana, Cameroon and Indonesia) for their effects on cytokine production (TNF-α, IL-1β, IL-6 and IL-10), using phorbol 12-myristate 13-acetate (PMA)-differentiated THP-1 cells stimulated with LPS. We then used enzyme-linked immunosorbent assays (ELISAs) for cytokine level assessments. The most effective propolis for modulating cytokine levels was then assessed by liquid chromatography-mass spectrometry (LC-MS)-based metabolomics to confirm whether or not metabolomics was an effective tool for elucidating the mechanism of action of the propolis.

## 2. Results

### 2.1. Cytotoxicity of Propolis Extracts against PMA-Differentiated THP-1 Cells

**Figure 1:**
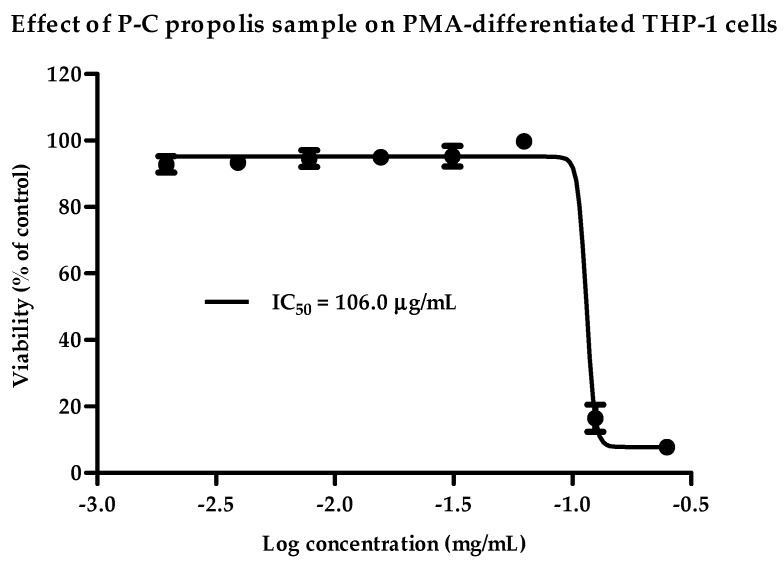
Cytotoxic effects of the Cameroon (P-C) propolis extract at varying doses on phorbol 12-myristate 13-acetate (PMA)-differentiated THP-1 cells. The P-C extract was cytotoxic to PMA-treated cells, with an IC50 of 106.0 µg/mL. Each data point represents the mean ± SD (*n* = 3).

Crude propolis extracts from samples collected from the UK (P-UK), Ghana (P-G), Cameroon (P-C) and Indonesia (P-Ind) were tested for cytotoxicity using assays performed on PMA-differentiated THP-1 cells (Figure 1 and Figure S1 in supplementary materials). Dose-response relationships were clearly observed, except with the P-G and P-Ind2 extracts. The P-UK1-5 extracts showed a slight dose effect on THP-1 cells, with a range of IC50 values between 46 and 58 µg/mL. The P-Ind1 extract was the most toxic and gave an IC50 value of 25.73 µg/mL. By contrast, P-Ind2 and P-C gave the lowest toxicity at >250 and 106 µg/mL, respectively (Table 1).

The level of secretion of four cytokines was analysed by ELISA following treatment of the cells with propolis extract. Table 1 shows the final selected propolis concentrations used for cytokine production assessments. The cells remained viable at these concentrations, which were below their respective IC50 Values. P-C extract was chosen as the best model extract to study the metabolic responses of propolis as an anti-inflammatory agent and was used at a final concentration of 70 µg/mL.

### 2.2. Effect of Propolis Extracts on Pro-Inflammatory TNF-α Cytokine Production

**Figure 2:**
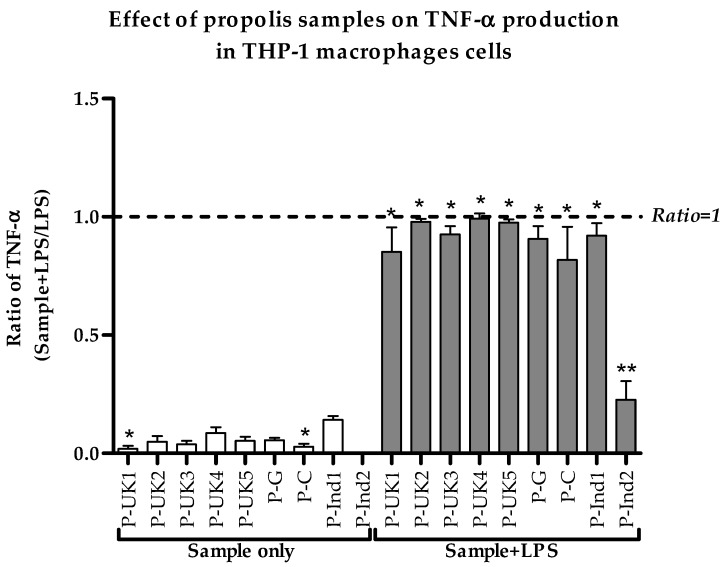
Effect of propolis extracts on the production of TNF-α by phorbol 12-myristate 13-acetate (PMA)-differentiated THP-1 cells in the absence and presence of lipopolysaccharide (LPS) (0.5 µg/mL). The TNF-α levels were significantly different from the negative control levels in all eight combination treatments, except for the P-Ind2 extract (*n* = 3). *: Significant (*p* < 0.05) when compared with untreated control; **: Significant (*p* < 0.05) when compared with LPS alone; P-UK (1–5): Five propolis extracts from the UK; P-G: Propolis from Ghana; P-C: Propolis from Cameroon; P-Ind (1 and 2): Two Propolis extracts from Indonesia.

The sample extracts on their own showed no effect on the production of pro-inflammatory TNF-α in THP-1 cells when compared to a negative untreated control. However, when the cells were stimulated with LPS, the extracts inhibited the secretion of TNF-α, compared with LPS alone (ratio < 1.0). Treatment with P-Ind2 extract greatly decreased the cytokine levels by approximately 80% and reached statistical significance when compared to LPS alone. Clear inhibitions of 15% and 20% were also observed with the P-UK1 and P-C extracts, respectively. As shown in Figure 2, the inhibitions were statistically significant when compared to negative control cells in all combination treatments with LPS, except for the P-Ind2 extract.

### 2.3. Effect of Propolis Extracts on Pro-Inflammatory IL-1β Cytokine Production

**Figure 3:**
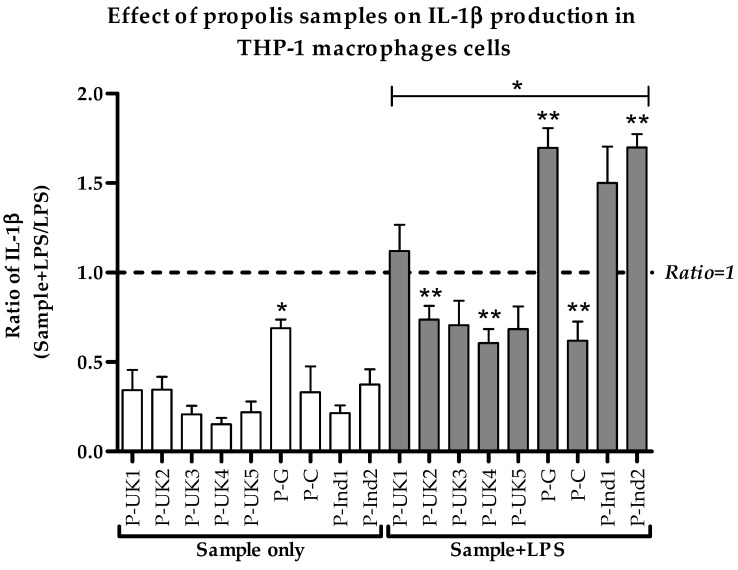
Effect of propolis extracts on the production of IL-1β by phorbol 12-myristate 13-acetate (PMA)-differentiated THP-1 cells in the absence and presence of LPS (0.5 µg/mL). All nine combined treatments were significantly different from the negative control cells (*n* = 3). LPS: Lipopolysaccharides; *: Significant (*p* < 0.05) when compared with untreated control; **: Significant (*p* < 0.05) when compared with LPS alone; P-UK (1–5): Five propolis extracts from the UK; P-G: Propolis from Ghana; P-C: Propolis from Cameroon; P-Ind (1 and 2): Two Propolis extracts from Indonesia.

An inhibition of secretion of the pro-inflammatory IL-1β was clearly observed for five of the propolis extracts (Figure 3). The P-UK4 and P-C extracts, in combination with LPS, gave the highest inhibitory effect on cytokine secretion, at about 40%, when compared with LPS alone (ratio < 1.0). In contrast to the TNF-α level, the levels of IL-1β were surprisingly enhanced by treatment with P-UK1, P-G, P-Ind1 and P-Ind2, at ~11%, ~70%, ~50% and ~70%, respectively, when the cells were co-stimulated by LPS. The ratios of secreted cytokines differed significantly in response to the combination treatments of LPS and propolis extracts when compared to those of the negative control cells. Cells treated with propolis extracts only showed a slight increase in the background level release of cytokine, and this was only significant (*p* < 0.05) with the P-G extract (Figure 3).

### 2.4. Effect of Propolis Extracts on Pro-Inflammatory IL-6 Cytokine Production

**Figure 4:**
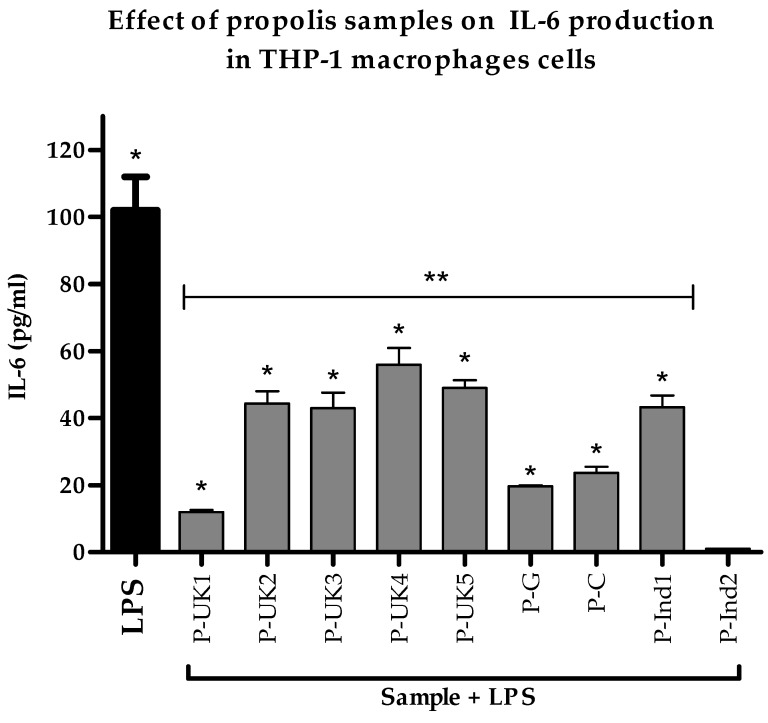
Effect of propolis extracts on the production of IL-6 by phorbol 12-myristate 13-acetate (PMA)-differentiated THP-1 cells in the absence and presence of LPS (0.5 µg/mL). All eight combined treatments apart from the P-Ind2 extract were significantly different from the negative and positive control (*n* = 3). LPS: Lipopolysaccharides; *: Significant (*p* < 0.05) when compared with untreated control; **: Significant (*p* < 0.05) when compared with LPS; P-UK (1–5): Five propolis extracts from the UK; P-G: Propolis from Ghana; P-C: Propolis from Cameroon; P-Ind (1 and 2): Two Propolis extracts from Indonesia.

The secretion of IL-6 produced by LPS-stimulated THP-1 cells in response to all propolis extracts was lower when compared to cells stimulated with LPS alone (Figure 4). The P-UK1 extract showed the greatest effect on the cytokine level; however, the P-Ind2 extract had no effect on the level of this cytokine from the background level. The concentrations of secreted IL-6 were significantly lower when compared to the concentrations secreted by the positive control LPS. Propolis extract alone either had no effect on the release of this cytokine or the release was undetectable (Table S3).

### 2.5. Effect of Propolis Extracts on Anti-Inflammatory IL-10 Cytokine Production

**Figure 5:**
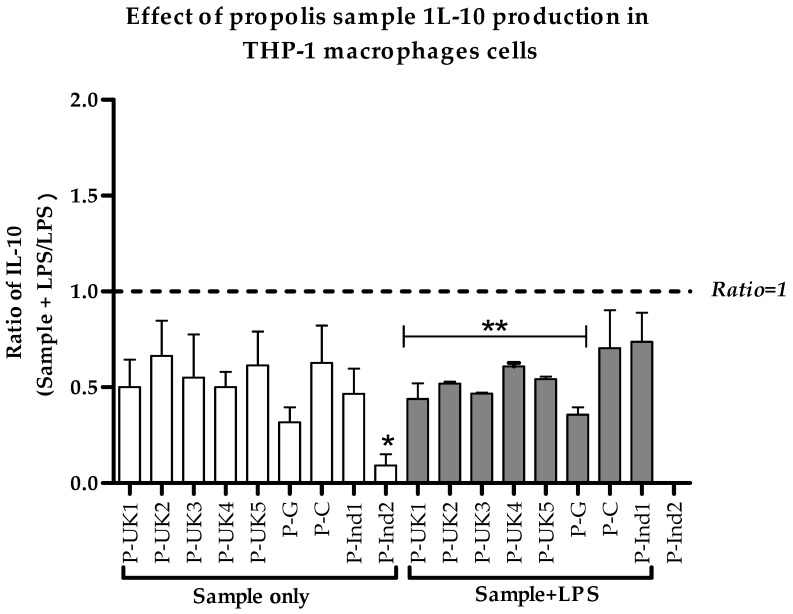
Effect of propolis extracts on the production of IL-10 cytokines by phorbol 12-myristate 13-acetate (PMA)-differentiated THP-1 cells in the absence and presence of LPS (0.5 µg/mL). P-UK1-5 and P-G combined treatments of propolis and LPS were statistically different from LPS alone (*n* = 3). LPS: Lipopolysaccharides; *: Significant (*p* < 0.05) when compared with untreated control; **: Significant (*p* < 0.05) when compared with LPS; P-UK (1–5): Five propolis extracts from the UK; P-G: Propolis from Ghana; P-C: Propolis from Cameroon; P-Ind (1 and 2): Two Propolis extracts from Indonesia.

The effect of propolis extracts on the secretion of the anti-inflammatory IL-10 cytokines by THP-1 cells was measured by adding the extracts on their own and in combination with 0.5 µg/mL LPS. Surprisingly, the levels of these cytokines were only slightly or negligibly affected following combined treatment with LPS when compared with LPS alone (Figure 5). The secretions were slightly altered from their negative control cells. The differences were not statistically significant from negative control cells; however, these negative effects could explain a more subtle mechanism of the anti-inflammatory action of propolis.

### 2.6. Effect of Cameroonian Propolis (P-C) on the Cell Metabolome

**Figure 6:**
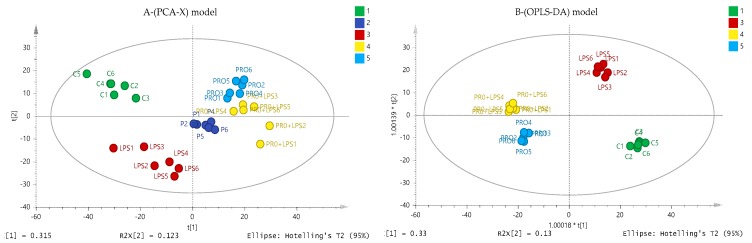
(**A**) PCA-X versus (B) OPLS-DA score plots of THP-1 cells. The figures show a clear separation between control, pooled and treatment groups (LPS, Propolis and Propolis+LPS) based on 403 polar metabolites separated on a ZIC-pHILIC column (*n* = 6). The PCA score plot (**A**) gives R2X = 0.583, Q2 = 0.409. The OPLS-DA score plot (**B**) gives R2X = 0.640, R2Y = 0.984, Q2 = 0.753. (C: Control; Pro: Cameroonian propolis (P-C) propolis extract; LPS: Lipopolysaccharides; Pro+LPS: Propolis and LPS (= P-C + LPS) combination treatments; P = pooled samples). PCA: Principle component analysis.

Understanding the mechanism underlying the anti-inflammatory response to each propolis extract would be worthwhile; however, since the P-C extract showed the highest activity on TNF-α, IL-1β and IL-6 cytokines levels, it was selected for further metabolomic evaluation of its anti-inflammatory action. Metabolomics profiling of the PMA-differentiated THP-1 cells following LPS, P-C and P-C+LPS treatments were examined to assess the antagonistic effect of the P-C extract to LPS. Principle component analysis (PCA) gave a perfect clustering of quality control samples in the middle of the Figure 6A (P 1-6). This indicates the precision and stability of the instruments during the sample runs on the ZIC-pHILIC column for all the polar metabolites. Comparison of the control, LPS, P-C alone and P-C+LPS groups showed clear group separations using an OPLS-DA model (Figure 6B). The separation between treatment and control groups would suggest different biochemical interactions of P-C+LPS compared with LPS alone.

The data set for all polar metabolites was filtered by excluding metabolites with relative standard deviation (RSD) values >20% within the pooled samples. The most significantly altered metabolites are summarised in Table 2. The LPS and propolis extract groups were compared to normal control cells (LPS/C and P-C/C, respectively), whereas the LPS-treated cells were compared to the combination treatments to give a clearer depiction of the effect of propolis alone and to explain the difference due to LPS effects (P-C+LPS/LPS). The aim was to confirm the distinct metabolic profile for the treatment with propolis in the presence and absence of LPS. The univariate analysis in Table 2 shows that different pathways were significantly changed, including glycolysis, the tricarboxylic acid (TCA) cycle, oxidative phosphorylation (OXPHOS), arginine and proline metabolism and purine and pyrimidine metabolism.

Ratio plot analysis (Figure S14) of the metabolomics data from THP-1 derived macrophages cells characterise the highest and lowest abundance and accumulated metabolites. Statistically significant differences between P-C+LPS and LPS were observed in 62 polar metabolites. It visualises the error associated with the ratio calculation for each metabolite (*n* = 6). Upregulation of most of the metabolites was observed clearly upon treatment with propolis extract. It was characterized that inflammatory-related metabolites, such as hypoxanthine, putative acetyl-CoA and citrate, were altered by the combination treatments.

## 3. Discussion

Macrophages, upon stimulation by various microbial and environmental signals, polarise into different subpopulations with distinct purposes. These cell subpopulations are crucial for the inflammatory process and for defence against infections, and they conduct these processes through the secretion of such molecules nitric oxide (NO) and inflammatory cytokines, such as TNF-α, IL-1β and IL-6 [26]. Over-secretion of these mediators has been observed in several inflammatory diseases and cancer [43]. The aim of the present study was to assess the use of propolis samples from different regions as anti-inflammatory agents.

Cytotoxicity assays were performed on THP-1 cells using nine different propolis extracts. The ethanolic extracts of the propolis showed different cytotoxicities towards the cells, which might be explained by their different chemical compositions. The sensitivity of the cells to propolis extracts from the UK (P-UK1-5) were very close to each other (Figure S1), with respect to their differences in cytokine responses. P-Ind1 showed the highest toxicity to THP-1 cells; however, the cells were still >90% viable at the dose chosen for cytokine assessments (15 µg/mL). Based on the cytokine production and anti-inflammatory performance of the propolis extracts, P-C was chosen for further metabolic investigations at 70 µg/mL. Among the assessed cytokines, pro-inflammatory IL-6 showed the strongest reduction in secretion in response to all propolis extracts at their respective selected concentrations (Table 1). The secretion of TNF-α was significantly decreased with P-UK1, P-C and P-Ind2 and only slightly decreased with the other extracts. P-UK4 and P-C showed the most pronounced anti-IL-1β effects. Interestingly, the secretion of this cytokine in LPS-stimulated THP-1 cells was enhanced by P-UK1, P-G, P-Ind1 and P-Ind2, suggesting the possibility that different mechanisms might exist for the secretion of each cytokine in LPS-stimulated macrophages [26].

Since P-C showed the most consistent effects in lowering the cytokine response, it was selected for further study. The metabolite responses were observed in THP-1 cells following administration of LPS, P-C and P-C+LPS with the goal of determining whether the metabolite alterations induced by propolis in the presence or absence of LPS could provide a better understanding of the mechanisms and pathways involved in the anti-inflammatory characteristics of propolis.

Cameroonian propolis has been chemically investigated and contains triterpenes [32] that have shown dose-dependent anti-inflammatory actions [44]. In addition, its biological activity might reflect the presence of many caffeic acid derivatives [45,46]. Propolis and its isolated compounds were also reported to decrease the release of inflammatory cytokines through suppression of NF-κB activation [47,48]. The NF-κB pathway can be activated by LPS through TLR recognition [49,50]. Blockade of cytokines, and particularly of IL-1 and TNF-α, in immune inflammatory diseases has provided the greatest advances in medicine and in the development of novel treatments for inflammation [51]. TNF-α plays a crucial role in initiating the cascade of pro-inflammatory cytokines and their subsequent inflammatory processes. IL-6 is also produced rapidly in acute inflammatory responses [26,51].

Several studies have shown that propolis has immunological activity [52]. Another study has shown that the antioxidant and anti-inflammatory activity of Brazilian green propolis in stimulated J774A.1 macrophages occurs by the inhibition of reactive oxygen species (ROS), NO and pro-inflammatory cytokines, including TNF-α, IL-1β and IL-6 [53]. In the present study, production of these cytokines was clearly inhibited by propolis extracts apart from P-UK1, P-G and P-Ind1 and 2 in IL-1β. We therefore sought to gain further insight into their immunosuppressive mechanism of action by conducting non-targeted metabolic profiling of the effect of Cameroonian propolis on THP-1 cells. Using the same method of metabolic identification, no trace of any metabolites was found in the P-C sample.

Activation of TLRs, particularly with LPS, leads to a switch from oxidative phosphorylation (OXPHOS) towards glycolysis in immune cells [54], similar to the response observed in tumour cells. Stimulation with LPS revealed significant changes in amino acid, carbohydrate and nucleotide metabolism [55]. In addition, clear differences were previously reported for the fat metabolome [55,56]. The results of the present study confirm the effect of LPS on macrophage cells and further examine anti-LPS activity by propolis extracts using a metabolomics approach.

While there appear to be effects of LPS on glycolysis, these are not that prominent and there is no marked effect of P-C in countering these. Taking the data as a whole, the very large effects on metabolite levels can be seen in purine metabolism. LPS produces a 14-fold increase in the level of hypoxanthine and treatment with P-C completely abolishes this and promotes a 13-fold increase in the precursor of hypoxanthine, inosine. P-C treatment on its own also produces a 10-fold increase in inosine. Thus, it would seem that P-C is acting as an inhibitor of purine nucleoside phosphorylase (PNP). This is an enzyme that can hydrolyse a range of nucleosides and the proposed mode of action is supported by accumulation of adenosine, guanosine and xanthosine, which are also substrates of the enzyme [57,58,59]. In addition, methylthioadenosine [60] and possibly S-adenosylmethionine are hydrolysed by a similar mechanism and these metabolites also accumulate. PNP deficiency causes severe immunosuppression in people who are deficient in the enzyme [61]. PNP-deficient mice were found to accumulate guanosine and inosine [62]. It is currently not completely known why PNP deficiency causes immunosuppression. Deficiency in purine nucleotide deaminase that converts adenosine monophosphate to inosine monophosphate (AMP to IMP) also causes immunosuppression. Our previous study examined the effect of LPS and a combination of LPS and melittin on THP-1 cells and identified an increase in the level of hypoxanthine [55]. Interestingly, in the current case, this increased level was suppressed by ~30% following treatment with P-C and LPS together, compared to LPS alone. One possible explanation for the effect on the immune response is that hypoxanthine is a major substrate for the production of superoxide via the action of xanthine oxidase [63,64] and impairment of superoxide production might impair the immune response. Coupled to the production of superoxide from hypoxanthine oxidation is indole dioxygenase (IDO), and this enzyme is responsible for degrading tryptophan via the kynurenine pathway [65,66]. This degradative pathway lowers the immune response through producing various molecules in the kynurenine pathway, including kynurenine, which can bind to the aryl hydrocarbon receptor (AHR) receptor causing immune suppression [67,68]. Regan et al. reported depletion in tryptophan with an accumulation of kynurenine following stimulation by LPS and IFN-α [69,70]. The anti-inflammatory drug indomethacin caused a significant attenuation of the effects of LPS on tryptophan and a reduction in the level of kynurenine was observed following IFN-α stimulation, but no effect on kynurenine was observed after LPS treatment [70].

In the current case, kynurenine levels are increased by both the LPS and P-C treatments and may contribute to immune-modulation. Thus, the production of hypoxanthine in the purine nucleotide cycle (PNC) could both simulate the immune response through acting as a substrate for xanthine oxidase, resulting in the production of superoxide, and also reduce it through promoting production of kynurenine.

IMP is formed as a result of an excessive demand for ATP, such as in skeletal muscle during intense exercise [71,72]. The high demand results in some of the ATP pool in the muscle being used up, and the AMP formed as a result is converted to IMP and enters the purine nucleotide cycle (PNC) with subsequent conversion into inosine and hypoxanthine that are lost from the muscle and enter the blood. It may be that the high demand for ATP in activated macrophages is analogous to the situation in skeletal muscle. IMP is recycled back to AMP via an enzymatic reaction involving aspartate and guanosine 5’-triphosphate (GTP), thus conserving purines and ultimately supporting the production of ATP; this process with regard to skeletal muscle and exercise is more efficient in trained individuals. In the THP macrophages, there is a marked increase in GTP resulting from the P-C treatment both with and without LPS and there is a corresponding rise in AMP in the presence of P-C. Associated with this, there are elevations in both ATP and ADP in the P-C-treated cells. The source of the elevated GTP cannot be exactly pinpointed from the metabolic profile. GTP can be formed in the tricarboxylic acid (TCA) cycle in the conversion of succinyl coenzyme A (succinyl CoA) to succinate; succinate levels are slightly higher in the P-C-treated samples. However, it is difficult to infer from the levels of metabolites in a cycle whether or not higher levels of a particular metabolite are due to increased flux through the cycle or accumulation due to slowing down. The recent literature indicates that the TCA cycle is disrupted by treatment of macrophages with LPS, resulting in an accumulation of succinate and the dicarboxylic acid itaconate [56]. In the current case, there was no evidence of itaconate accumulation. Significant increases in pyruvate with acetyl-CoA reduction by LPS are supported by several previous studies [55,56], which also support the shift towards lactate rather than acetyl-CoA production. The combination of LPS with P-C antagonised this effect and reversed the effects of LPS pyruvate and acetyl-CoA so that they were significantly decreased and increased, respectively. An increase in putative acetyl-CoA would favour an increased flux towards the TCA cycle, as detected here, which would, in turn, enhance the formation of citrate and oxaloacetate. The citrate is converted to acetyl-CoA and oxaloacetate via the ATP-citrate lyase enzyme in the presence of ATP. Acetyl-CoA helps activated macrophages to meet their lipid biosynthesis and biosynthetic demands [56]. At the same time, citrate enhances the production of NO and ROS through the conversion of oxaloacetate to pyruvate and nicotinamide adenine dinucleotide phosphate (NADPH). The latter generates ROS by NADPH oxidase and is used for NO and citrulline production from L-arginine [73]. This also would explain the increased production of the putative acetyl-CoA and oxaloacetate reported in this study in response to LPS alone (Table 2). However, these two metabolites were increased significantly in LPS and P-C combination treatment, which supressed LPS activity.

An increase in the levels of NADH in the P-C-treated samples also supports the proposal that the treatment is promoting the TCA cycle. Increased flux through the TCA cycle could support the increase in levels of ATP and GTP resulting from the propolis treatments. Increases in ATP would support the immunological response; thus, the propolis as a complex mixture seems to have more than one mode of action and overall the effect is immunomodulatory, both supporting the immune response and decreasing it as evidenced by the decreased release of cytokines. Overall, the P-C treatment increases the high-energy phosphates, which are derived from ATP, in the cells with cytidine triphosphate (CTP), uridine mono- and di-phosphate (UMP and UDP) levels also increasing in the presence of P-C. UTP levels are decreased by the P-C treatment, but this might be due to the UTP being consumed in producing increased levels of the UDP conjugates with glucose, xylose and N-acetyl glucosamine. These conjugates are employed for the biosynthesis of glycan chains attached to cell-surface proteins. It has been proposed that increases in these conjugates occur when the macrophages polarise towards their M2 phenotype, where glycation of receptors may be responsible for a decrease in response to LPS [74,75].

Both L-citrulline and NO are by-products of arginine metabolism by the inducible nitric oxide synthase (iNOS) [76], and citrulline levels were significantly increased in response to LPS (Table 2). The production of citrulline was also elevated by propolis alone; however, citrulline production was not significantly different in cells treated with a combination of P-C and LPS when compared to LPS alone and was accompanied by a significant increase in its putative product metabolite N-(L-arginino) succinate, which is required to recycle citrulline back into arginine. Ethanolic extracts of propolis significantly and dose-dependently inhibited the production of NO in macrophage cells [53]. The ability to sustain NO generation plays a crucial role in macrophage adaptation that allows for killing of intracellular mycobacteria [77,78]. Moreover, IL-1β cytokines also have an important role in infected macrophages [79]. Conversely, continuous production of NO from iNOS activation inhibits IL-1β through the inflammasome [80]. Thus, an environment-dependent adjustment of macrophage function might be activated through the activity of the arginine, including pathways such as the citrulline-arginine cycle and argininosuccinate pathway [81].

The antagonism of LPS by the LPS+P-C combination treatment was also observed in this study through the increase in NADP+ and a decrease in pyruvate level, which would prevent further ROS and NO production. Furthermore, the TCA cycle is interrupted by LPS through the inhibition of succinate dehydrogenase (SDH), causing an elevation in the succinate level [82,83]. The elevation in succinate in response to LPS alone promotes inflammation by inhibiting prolyl hydroxylase (PHD) activity and subsequent accumulation of the hypoxia induced factor-1α (HIF-1α) protein [82]. SDH is an integral component of the respiratory chain complex II; its inhibition by the LPS leads to a reduction in mitochondrial respiration [73]. The activity of complex I in the mitochondria can also be supressed by the accumulated succinate, which causes further ROS production [81,84].

Several previous studies have described the reprogramming of macrophage cells upon stimulation, particularly with respect to glucose-PPP (pentose phosphate pathway) metabolism [85]. In M1 macrophages, LPS downregulates carbohydrate kinase-like protein (CARKL), leading to a high cellular redox state [86]. This would lead to a build-up of several metabolites [76,86], including putative sedoheptulose-7-phosphate (S7P), ribose-5-phosphate and fructose-6-phosphate, which increased significantly in response to LPS (Table 2). Cell reprogramming occurs to refocus the macrophage in order to increase PPP and glycolysis to support M1 polarisation [85]. However, a general increase was observed in the present study in the level of glycolysis, TCA cycle, OXPHOS and PPP by the effect of the P-C and LPS combination treatment. Consequently, the levels of ADP, ATP, NADP+ and nicotinamide adenine dinucleotide (NADH and NAD+) were enhanced by the combination of P-C and LPS compared to LPS alone (Figure S14).

## 4. Materials and Methods

### 4.1. Extract Preparation

Nine propolis samples from the UK (P-UK1-5), Ghana (P-G), Cameroon (P-C) and Indonesia (P-Ind1-2) were extracted. Ethanol extracts of approximately 10 g propolis were prepared by vigorous mixing and sonication for 60 min using a sonicating bath (Fisher Scientific, Loughborough, UK). The extracts were filtered and the propolis was re-extracted twice with 100 mL ethanol (Fisher Scientific, Loughborough, UK). The extracts were combined and evaporated, and the residue was stored at room temperature until required for the assays.

### 4.2. Cell Culture and Differentiation

The THP-1 cell line was obtained from American Type Culture Collection-ATCC^®^ (Porton Down, Salisbury, UK) and maintained at a 1 × 10^5^ cells/mL seeding density in RPMI 1640 (Thermo Fisher Scientific, Loughborough, UK) containing 10% (*v*/*v*) foetal calf serum (FCS) (Life Tech, Paisley, UK), 2 mmol/L L-glutamine (LifeTech, Paisley, UK) and 100 IU/100 µg/mL penicillin/streptomycin (Life Tech, Paisley, UK). Cells were sub-cultured using fresh media every 2–4 days and maintained in an incubator (37 °C, 5% CO_2_, 100% humidity). THP-1 cells were differentiated using PMA (Sigma-Aldrich, Dorest, UK) at a final concentration of 60 ng/mL and incubated for 48 h. THP-1 cell differentiation was enhanced by removing the PMA-containing media and adding fresh media for a further 24 h. Cells were checked under a light microscope for the evidence of differentiation.

### 4.3. Cell Viability Assay

The THP-1 cells were seeded at a density of 1 × 10^5^ cells/well in 96-well plates and incubated for 24 h at 37 °C in a humidified atmosphere of 5% CO_2_. After 24 h, the cells were treated with different concentrations of propolis samples (2.0–250 µg/mL) and incubated for a further 24 h. Untreated control cells and medium were added to the plates and dimethyl sulphoxide (DMSO) was used as a positive control. Resazurin salt solution (0.1 mg/mL) was added at a final concentration of 10% (*v*/*v*) and the plates were incubated for a further 24 h. Fluorescence readings were taken using a SpectraMax M5 plate reader (Molecular Devices, Sunnyvale, CA, USA) at λEx of 560 nm and λEm of 590 nm. After background correction, cell viability for each concentration was calculated relative to the mean value of negative control (*n* = 3). GraphPad Prism for Windows (version 5.00, GraphPad Software, San Diego, CA, USA) was used to obtain dose–response curves and mean inhibitory concentration (IC50) values.

### 4.4. Cytokine Production

After 48 h of differentiation using PMA (60 ng/mL) in 24-well plates, the media were aspirated, and the cells were incubated for a further 24 h in PMA-free medium. At day 4, the cells were incubated with final concentrations of propolis samples (Table 1) with and without LPS (Sigma-Aldrich) (0.5 µg/mL) for an additional 24 h. Conditioned medium was collected and frozen until required for ELISA (*n* = 3).

### 4.5. Enzyme-Linked Immunosorbent Assay (ELISA)

ELISA Ready-Set-Go kits were purchased from Thermo Fisher Scientific (Loughborough, UK). The assays were performed according to the manufacturer’s instructions to quantify the release of inflammatory cytokines (TNF-α, IL-1β, IL-6 and IL-10). The reaction was stopped using acid solution (2 N sulphuric acid). The plates were read using a SpectraMax M5 plate reader (Molecular Devices, Sunnyvale, CA, USA) at 560 nm and the absorbance values were corrected by subtracting readings taken at 570 nm.

### 4.6. Metabolite Extraction

The PMA-differentiated THP-1 cells were grown for 48 h in 6-well plates seeded at a density of 4.5 × 10^5^ cells/well (*n* = 6). The medium was aspirated and replaced with fresh medium for a further 24 h, and then the cells were incubated with LPS, P-C, or a combination of LPS and P-C for an additional 24 h. The final concentrations of LPS and P-C were 0.5 and 70 µg/mL, respectively. After 24 h, the medium was aspirated, and the cells were washed with 3 mL of phosphate-buffered saline (PBS) (Sigma-Aldrich) at 37 °C. The cells were extracted (1 mL per 1 × 10^6^ cells) by ice cold extraction solution (methanol:acetonitrile:water, 50:30:20 (*v*/*v*), containing 5 µg/mL of internal standard 13C2 glycine (Sigma-Aldrich, Poole, UK)). The cells were scraped, and cell lysates were mixed in a Thermomixer (12 min, 4 °C), and then centrifuged for 15 min at 0 °C (13,500 rpm). The supernatants were collected and stored at −80 °C until required for LC-MS analysis. The stability and reproducibility of the analytical method were ensured by injecting authentic standard metabolite mixtures and quality control (QC) samples throughout the runs. The analytical standards were prepared by adding 10 µg/mL final concentration of each metabolite standard [87] containing 13C2 glycine, distributed into seven different standard solutions. The pooled quality control samples were prepared by pipetting 20 µL from each of the samples and mixing them together before transferring them into a HPLC vial.

### 4.7. LC-MS Conditions

An Accela HPLC system interfaced to an Exactive Orbitrap mass spectrometer (Thermo Fisher Scientific, Bremen, Germany) was used for the liquid chromatographic separations. ZIC-pHILIC (150 × 4.6 mm, 5 µm) HPLC columns supplied by HiChrom (Reading, UK) were used. Samples were run on LC-MS under the following conditions: the ZIC-pHILIC mobile phase consisted of 20 mM ammonium carbonate in HPLC-grade water (A) and acetonitrile (B); the solvent gradient used was 80% B (0 min), 20% (30 min), 8% (31–36 min), and 80% (37–45 min) at a flow rate of 0.3 mL/min. The nitrogen sheath and auxiliary gas flow rates were maintained at 50 and 17 arbitrary units. The electrospray ionisation (ESI) interface was employed in a positive/negative dual polarity mode, with a spray voltage of 4.5 kV for positive mode and 4.0 kV for negative mode, while the ion transfer capillary temperature was set at 275 °C. Full scan data were obtained in the mass-to-charge ratio (*m*/*z*) between 75 and 1200 amu for both ionisation modes. The data were collected and processed using Xcalibur 2.1.0 software (Thermo Fisher Scientific, Bremen, Germany).

### 4.8. Data Extraction and Statistical Analysis

The data were extracted using MZMatch software (SourceForge, La Jolla, USA), http://mzmatch.sourceforge.net/). A macro-enabled Excel Ideom file was used to filter, compare and identify the metabolites (http://mzmatch.sourceforge.net/ideom.php). The metabolite lists obtained from these searches were then carefully evaluated manually by considering the quality of their peaks and the metabolites were matched with the retention times of authentic standards mixtures run in the same sequences. Library searches were also used for identification and carried out against accurate mass data of the metabolites in the Human Metabolome Data Base and KEGG (Kyoto Encyclopedia of Genes and Genomes). All metabolites were within 3 ppm of their exact masses. Univariate comparisons were performed using Microsoft Excel and paired t-tests between treated and control cells and differences were considered significant at *p* < 0.05. SIMCA-P software v.14.0 (Umetrics, Umea, Sweden) was used for multivariate analysis of the metabolite data by fitting PCA-X and OPLS-DA.

## 5. Conclusions

The present study addressed the main features involved in LPS activation of THP-1-derived macrophage cells and the possible use of propolis extracts as an anti-inflammatory treatment. The study results suggested that key pathways are perturbed by propolis in macrophage cells. Suppression of the LPS effects on cytokine production in these cells was confirmed for propolis samples from different regions. The antagonistic effects were detected in the levels of TNF-α, IL-1β and IL-6 pro-inflammatory cytokines. Although P-UK1, P-G and P-Ind1-2 extracts inhibited TNF-α and IL-6, they produced a stimulatory effect on IL-1β release. However, the P-C samples supressed the release of all four cytokines. Further study using a metabolic approach identified metabolic alterations that may contribute to the subsequent anti-inflammatory events. Both LPS and propolis changed the levels of metabolites in several different pathways, but the really major shifts in levels were in purine metabolism. The accumulation of several substrates of PNP in the presence of the P-C both with and without LPS being present suggested that it was acting as an inhibitor of PNP. Deficiency in PNP is associated particularly with a deficiency in T-cell immunity [88]. In addition, metabolic reprogramming by LPS caused an enhanced production of glycolysis and PPP products to replenish the disrupted TCA cycle and maintain ATP generation. Upregulation of some intermediate metabolites within glycolysis, the TCA cycle, oxidative phosphorylation and PPP in response to the combination of P-C and LPS were observed to counteract LPS activity. This metabolic investigation revealed the complexity of the macrophage responses to different treatments. Taken together, these data support several previous studies that suggest that propolis has clinical potential as a natural anti-inflammatory agent. Although a complex mixture varying in composition, propolis has a remarkably consistent biological effect that is possibly due to the selection pressure on bees causing collection of a material that provides similar biological properties regardless of its composition.

## Figures and Tables

**Table 1:** Regions where propolis was collected and the concentrations of different propolis samples used on phorbol 12-myristate 13-acetate (PMA)-differentiated THP-1 cells.

Propolis Samples		IC50 (µg/mL)	Selected Final Concentration (µg/mL)
Region	Extract Code		
**United Kingdom**	P-UK1	57.95	30
	P-UK2	48.99	15
	P-UK3	53.55	20
	P-UK4	48.08	15
	P-UK5	46.28	15
**Ghana**	P-G	86.95	15
**Cameroon**	P-C	106	70
**Indonesia**	P-Ind1	25.73	15
	P-Ind2	>250	250

**Table 2:** Significantly changed metabolites in THP-1 cells treated with either lipopolysaccharide (LPS) or Cameroonian propolis (P-C) extract alone in comparison with untreated controls and in cells treated with a combination of P-C and LPS compared with LPS treated cells. The results show the majority of affected metabolites. (0.5 µg/mL LPS; 70 µg/mL P-C).

Mass	Rt	Putative Metabolite	LPS/C		P-C/C		(P-C+LPS)/LPS	
			Ratio	*p*-Value	Ratio	*p*-Value	Ratio	*p*-Value
	**Arginine and Proline Metabolism**		-	-	-	-	-	-
145.08	15.39	4-Guanidinobutanoate	1.991	<0.001	1.549	0.002	1.419	0.002
115.06	13.01	l-Proline *	1.137	0.046	1.467	<0.001	1.303	0.001
113.06	11.14	Creatinine	0.884	ns	1.112	ns	1.454	0.030
175.10	16.04	l-Citrulline *	1.790	<0.001	2.148	<0.001	1.090	ns
129.09	16.02	4-Guanidinobutanal	1.494	0.032	1.723	<0.001	1.062	ns
290.12	16.78	N-(l-Arginino)succinate	0.556	0.001	0.919	ns	1.278	0.020
189.06	13.95	N-Acetyl-l-glutamate *	0.662	<0.001	3.983	<0.001	4.955	<0.001
145.16	26.37	Spermidine *	1.198	ns	0.169	<0.001	0.078	<0.001
		**Glycolysis/TCA cycle**						
260.03	16.83	d-Glucose 1-phosphate *	0.996	ns	0.531	<0.001	0.476	<0.001
260.03	15.95	d-Fructose 6-phosphate *	1.424	0.001	1.486	0.001	1.229	0.046
340.00	18.05	d-Fructose 1,6-bisphosphate *	2.242	<0.001	1.163	0.006	0.754	0.003
170.00	16.00	d-Glyceraldehyde 3-phosphate *	0.717	0.001	1.899	<0.001	1.622	<0.001
260.02	17.72	d-Glucose 6-sulfate	1.339	0.004	1.461	<0.001	1.118	ns
167.98	17.45	Phosphoenolpyruvate	1.254	ns	2.503	0.001	2.062	0.004
88.02	7.67	Pyruvate *	1.609	0.007	0.916	ns	0.483	<0.001
809.13	12.28	Acetyl-CoA	0.681	0.002	1.678	<0.001	1.912	0.001
190.01	15.80	Oxalosuccinate	0.588	0.044	5.385	ns	1.691	ns
132.01	15.74	Oxaloacetate *	0.700	0.041	1.064	ns	1.566	0.016
192.03	18.08	Citrate *	1.453	0.002	1.636	<0.001	1.214	0.040
118.03	14.94	Succinate *	1.323	0.005	1.534	<0.001	1.182	ns
116.01	14.92	Fumarate	0.894	ns	0.752	0.001	0.842	ns
131.07	14.89	Creatine *	0.647	<0.001	1.179	0.011	1.544	<0.001
427.03	15.19	ADP *	0.814	0.041	3.140	<0.001	2.425	<0.001
443.02	18.08	GDP *	0.727	0.003	1.917	<0.001	2.158	<0.001
507.00	16.55	ATP *	0.776	0.019	1.341	0.022	1.303	0.004
522.99	19.50	GTP *	0.923	ns	1.309	0.002	1.237	0.016
665.12	13.29	NADH *	0.757	0.004	1.623	<0.001	1.620	0.001
663.11	14.24	NAD+ *	0.363	<0.001	0.877	ns	1.893	<0.001
	**Oxidative Stress/ Pentose Phosphate Pathway**							
276.02	17.61	6-Phospho-d-gluconate *	1.355	0.001	0.521	<0.001	0.273	<0.001
196.06	13.15	d-Gluconic acid *	0.889	0.006	1.539	<0.001	1.310	<0.001
177.94	15.81	Pyrophosphate	1.271	0.009	1.406	<0.001	1.189	0.024
290.04	16.09	d-Sedoheptulose 7-phosphate	1.367	<0.001	1.970	<0.001	1.570	<0.001
370.01	18.21	d-Sedoheptulose 1,7-bisphosphate	1.900	<0.001	1.757	<0.001	0.893	ns
210.07	14.03	Sedoheptulose	1.741	0.002	1.202	ns	0.931	ns
230.02	15.68	d-Ribose 5-phosphate *	1.572	0.036	1.018	ns	0.736	ns
307.08	14.22	Glutathione	0.579	<0.001	1.508	<0.001	1.856	<0.001
612.15	17.27	Glutathione disulphide *	0.838	0.017	3.157	<0.001	3.791	<0.001
745.09	16.87	NADPH	0.494	<0.001	00.00	<0.001	00.00	<0.001
743.08	16.68	NADP+ *	0.896	ns	2.267	<0.001	2.063	<0.001
		**Purine Metabolism**						
347.06	15.61	dGMP	0.461	<0.001	1.575	0.001	1.937	<0.001
267.10	9.35	Adenosine *	1.276	ns	3.435	<0.001	2.445	<0.001
283.09	12.80	Guanosine *	1.677	ns	4.070	<0.001	3.202	0.004
268.08	11.06	Inosine *	2.967	<0.001	10.511	<0.001	13.160	<0.001
284.08	10.51	Xanthosine	2.064	ns	2.378	0.005	1.002	ns
251.10	7.93	Deoxyadenosine	2.912	<0.001	0.399	<0.001	0.292	<0.001
152.04	11.30	Xanthine *	1.766	0.002	1.772	<0.001	1.013	ns
168.03	12.34	Urate *	1.752	0.008	1.897	0.000	1.135	ns
136.04	10.41	Hypoxanthine *	14.695	<0.001	2.277	0.009	0.714	0.013
398.14	16.78	S-Adenosyl-L-methionine *	1.237	ns	2.578	<0.001	1.695	<0.001
297.09	16.78	5’-Methylthioadenosine *	0.700	0.001	2.727	<0.001	1.570	<0.001
348.05	15.39	IMP *	2.815	<0.001	3.341	<0.001	1.168	ns
347.06	13.75	AMP *	0.558	<0.001	2.164	<0.001	2.080	<0.001
		**Pyrimidine Metabolism/Glycan Chain Formation**						
125.06	10.94	5-Methylcytosine	1.486	0.004	2.124	<0.001	1.396	0.003
482.98	18.68	CTP *	0.603	<0.001	0.894	ns	1.403	<0.001
244.07	12.14	Pseudouridine	1.199	0.038	1.914	<0.001	1.668	<0.001
323.05	15.42	CMP *	0.742	0.002	1.915	<0.001	1.760	<0.001
111.04	11.13	Cytosine*	0.250	<0.001	0.359	0.003	1.142	ns
114.04	14.89	5,6-Dihydrouracil	0.631	<0.001	1.150	ns	1.604	<0.001
259.05	11.93	Glucosamine 1-phosphate	1.202	ns	1.711	0.001	1.360	0.020
324.04	15.06	UMP *	0.630	<0.001	2.223	<0.001	1.878	<0.001
404.00	16.48	UDP *	0.433	<0.001	2.482	<0.001	2.412	<0.001
483.97	17.79	UTP *	0.591	0.000	0.669	0.002	0.954	ns
566.05	16.04	UDP-glucose *	0.503	<0.001	0.955	ns	1.711	<0.001
536.04	16.07	UDP-d-xylose	0.666	0.002	2.042	<0.001	2.321	<0.001
580.03	18.83	UDP-glucuronate	0.583	<0.001	1.426	0.001	1.706	<0.001
607.08	14.91	UDP-N-acetyl-d-glucosamine *	0.579	<0.001	0.984	ns	1.539	0.001
		**Tryptophan Metabolism**						
220.09	9.74	5-Hydroxy-l-tryptophan isomer	8.133	<0.001	1.285	0.014	0.228	<0.001
191.06	10.29	5-Hydroxyindoleacetate *	1.527	<0.001	0.869	0.035	0.503	<0.001
219.05	10.31	5-Hydroxyindolepyruvate	1.515	<0.001	0.872	0.015	0.523	<0.001
117.06	11.07	Indole *	1.723	0.011	1.565	0.002	0.987	ns
204.09	11.85	l-Tryptophan *	1.182	ns	1.849	<0.001	1.531	0.013
208.09	11.09	l-Kynurenine *	1.835	0.001	1.591	0.001	0.810	ns
		**Miscellaneous**						
131.06	14.61	N-Acetyl-beta-alanine	1.306	<0.001	1.521	<0.001	1.263	0.011
210.04	17.19	d-Glucarate	0.611	0.005	1.385	0.020	2.367	<0.001
161.11	13.50	l-Carnitine *	0.899	ns	1.267	<0.001	1.504	0.002
149.05	11.72	l-Methionine *	1.380	0.011	1.527	<0.001	1.230	0.037
175.05	14.17	N-Acetyl-l-aspartate *	0.625	<0.001	1.117	ns	1.721	<0.001
203.12	11.26	O-Acetylcarnitine *	0.945	ns	3.291	<0.001	3.114	<0.001
226.11	15.85	Carnosine *	1.166	ns	1.558	<0.001	1.279	0.025
119.06	14.52	l-Threonine	1.432	<0.001	1.558	<0.001	1.140	ns
105.04	15.85	l-Serine *	1.432	0.008	1.625	<0.001	1.194	ns

Rt: Retention time (min); LPS: Lipopolysaccharide; P-C: Cameroonian propolis; P-C + LPS: combination treatment; *: Matches the analytical standard retention time; ns: non-significant.

## Supplementary Materials

The following are available online at https://www.mdpi.com/2218-1989/9/4/75/s1, Figure S1: Cytotoxic Effect of propolis samples on THP-1 cells, Figure S2–S13: Showing a representative 4-parameter logistic plot of TNF-α, IL-1β, IL-6, and IL-10 standard samples, Figure S14: The log2- fold change between P-C+LPS and LPS alone in THP-1 cells, Table S1-S4: Effect of propolis samples on the production of TNF-α, IL-1β, IL-6, and IL-10 cytokines in the presence and absence of LPS, Table S5: List of abbreviations, Table S6: List of catalog/serial numbers of instruments and reagents.
