# Supplementary material for: Propolis Exerts an Anti-Inflammatory Effect on PMA-Differentiated THP-1 Cells via Inhibition of Purine Nucleoside Phosphorylase
Source: Metabolites. 2019 Apr 16;9(4):75. doi: 10.3390/metabo9040075 (PMC6523283; doi:10.3390/metabo9040075)
Supplement: Supplementary file 1 [file metabolites-09-00075-s001.pdf]

Propolis Exerts an Anti-inflammatory Effect on PMA-Differentiated THP-1 Cells via Inhibition of Purine Nucleoside Phosphorylase

Abdulmalik M. Alqarni, Kanidta Niwasabutra, Muhamad Sahlan, Hugo Fearnley, James Fearnley, Valerie A. Ferro and David G. Watson

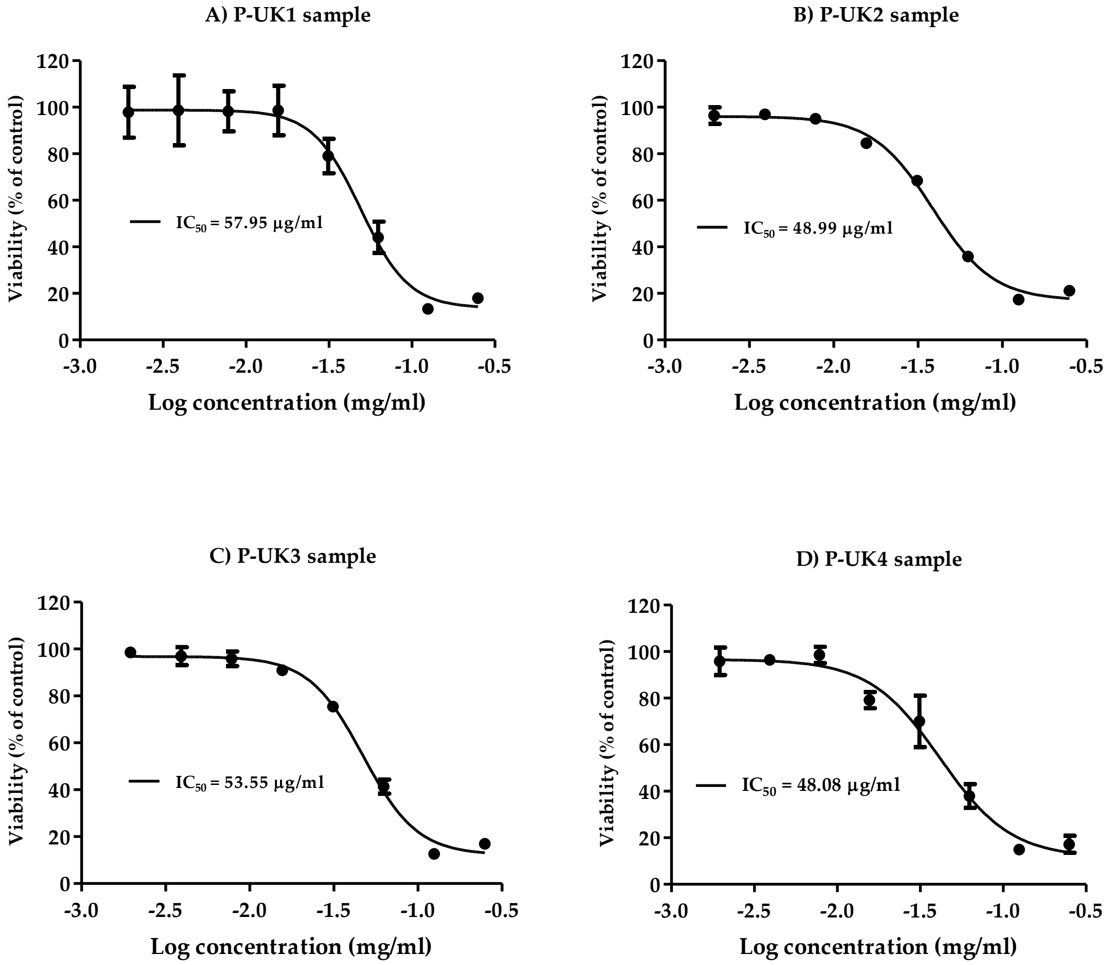

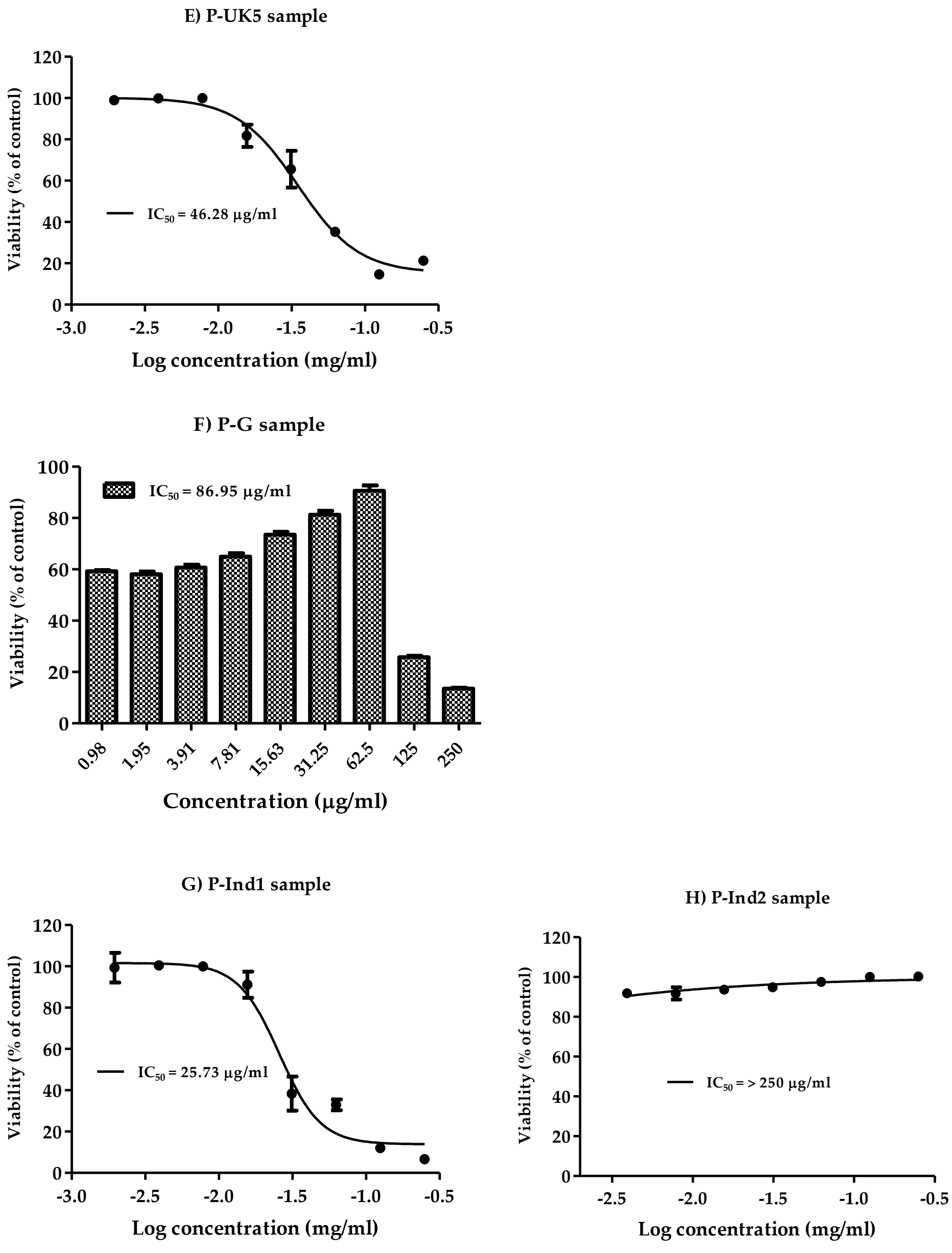

Figure S1: Cytotoxic effects of propolis extracts at varying doses on phorbol 12-myristate 13-acetate (PMA)-differentiated THP-1 cells. Each data point represents the mean ± SD (*n*=3).

P-UK (1-5): Five propolis extracts from the UK; P-G: Propolis from Ghana; P-Ind (1 and 2): Two Propolis extracts from Indonesia.

TNF-α production

Table S1: Effect of propolis extracts on the production of TNF-α cytokines in the presence and absence of LPS on PMA-differentiated THP-1 cells (n=3).

	TNF-α concentration (pg/ml)									
	**Sample only**					**Sample + LPS**				
**Propolis Samples**	n=1	n=2	n=3	Mean	RSD	n=1	n=2	n=3	Mean	RSD
**P-UK1**	21	2	94	39.00	124.54	981	1796	2093	1623.33	35.47
**P-UK2**	31	52	215	99.33	101.40	1526	1824	2112	1820.67	16.09
**P-UK3**	60	23	138	73.67	79.69	1304	1817	2089	1736.67	22.95
**P-UK4**	136	79	277	164.00	62.15	1573	1838	2121	1844.00	14.86
**P-UK5**	103	39	158	100.00	59.56	1519	1788	2136	1814.33	17.05
**P-G**	110	72	124	102.00	26.38	1218	1805	2104	1709.00	26.37
**P-C**	80	14	50	48.00	68.84	816	1813	2085	1571.33	42.52
**P-Ind1**	214	212	373	266.33	34.69	1237	1821	2141	1733.00	26.45
**P-Ind2**	<2.0	<2.0	<2.0	n/a	n/a	369	711	233	437.67	56.27
**Media**	113	132	196	147.00	29.58					
**LPS**	1521	1885	2183	1863.00	17.80					

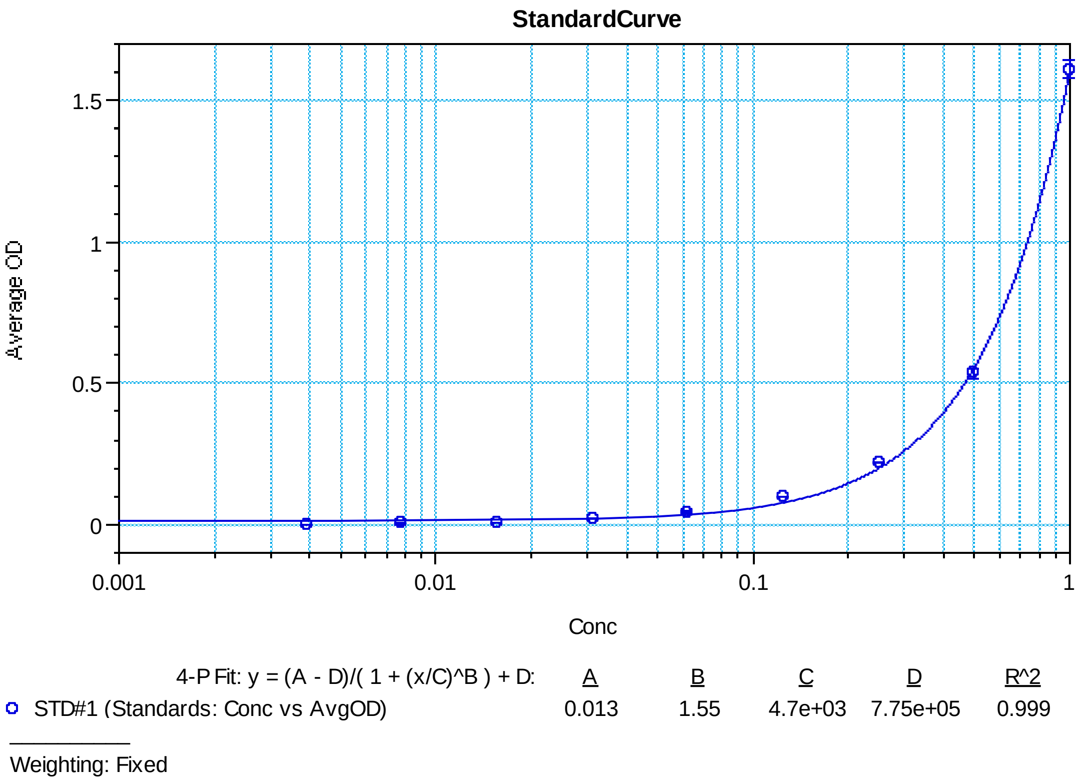

Figure S2: A representative 4-parameter logistic plot of TNF-α standard samples of 9 points showing the values of a, b, c, and d constants and the calibration equation with a perfect fit (R2=0.999). The data represents the mean ± SD of optical density (OD) values for duplicate standard concentrations (n=2).

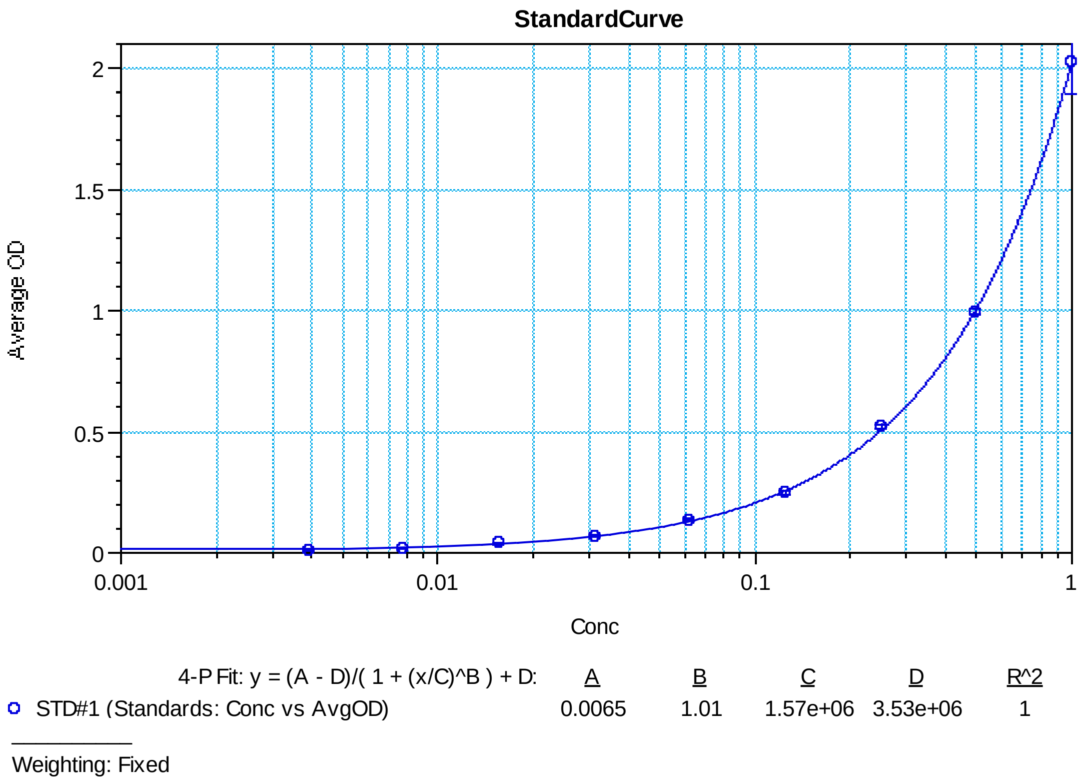

Figure S3: A representative 4-parameter logistic plot of TNF-α standard samples of 9 points showing the values of a, b, c, and d constants and the calibration equation with a perfect fit (R2=1.0). The data represents the mean ± SD of optical density (OD) values for duplicate standard concentrations (n=2).

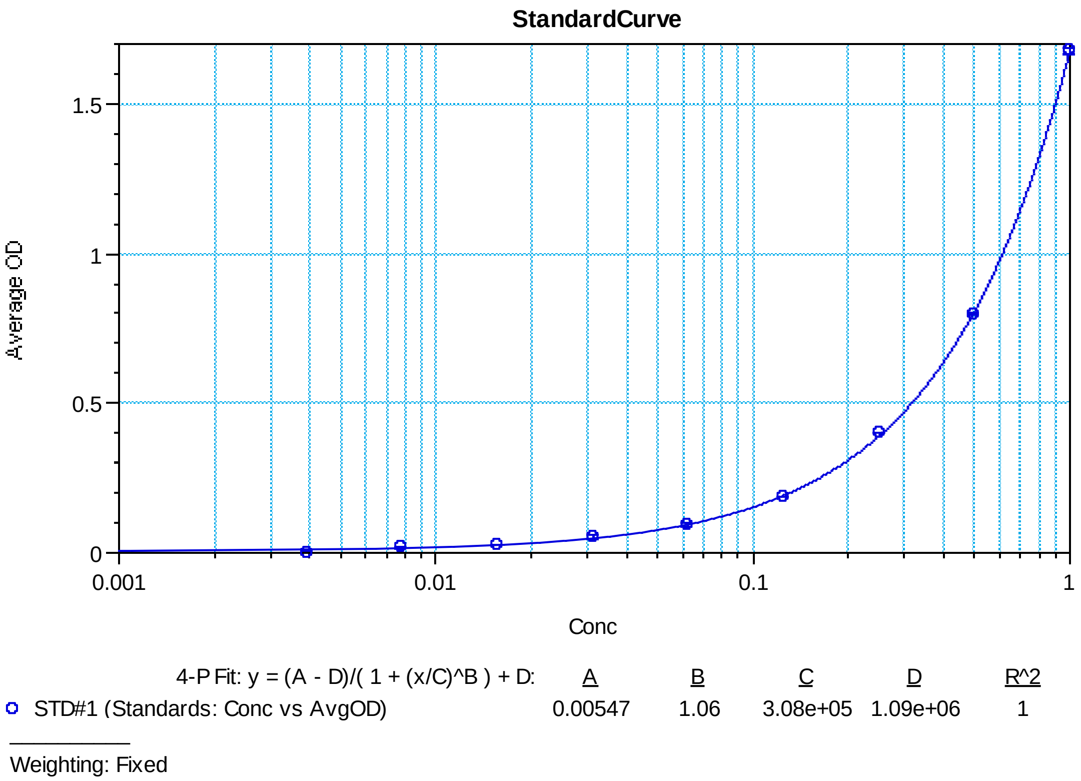

Figure S4: A representative 4-parameter logistic plot of TNF-α standard samples of 9 points showing the values of a, b, c, and d constants and the calibration equation with a perfect fit (R2=1.0). The data represents the mean ± SD of optical density (OD) values for duplicate standard concentrations (n=2).

IL-1β production

Table S2: Effect of propolis extracts on the production of IL-1β cytokines in the presence and absence of LPS on PMA-differentiated THP-1 cells (n=3).

	IL-1β concentration (pg/ml)									
	**Sample only**					**Sample + LPS**				
**Propolis Samples**	n=1	n=2	n=3	Mean	RSD	n=1	n=2	n=3	Mean	RSD
**P-UK1**	19.00	33.00	10.00	20.67	56.08	59.00	58.00	89.00	68.67	25.65
**P-UK2**	28.00	21.00	14.00	21.00	33.33	36.00	52.00	47.00	45.00	18.19
**P-UK3**	7.00	17.00	14.00	12.67	40.51	29.00	41.00	60.00	43.33	36.07
**P-UK4**	5.00	12.00	11.00	9.33	40.56	27.00	42.00	42.00	37.00	23.41
**P-UK5**	8.00	20.00	12.00	13.33	45.83	28.00	41.00	57.00	42.00	34.59
**P-G**	36.00	46.00	44.00	42.00	12.60	110.00	89.00	112.00	103.67	12.29
**P-C**	14.00	37.00	9.00	20.00	74.67	50.00	31.00	32.00	37.67	28.39
**P-Ind1**	9.00	18.00	12.00	13.00	35.25	79.00	77.00	120.00	92.00	26.38
**P-Ind2**	27.00	28.00	13.00	22.67	37.00	93.00	108.00	110.00	103.67	8.96
**Media**	6.00	10.00	16.00	10.67	5.03					
**LPS**	60.00	60.00	63.00	61.00	1.73					

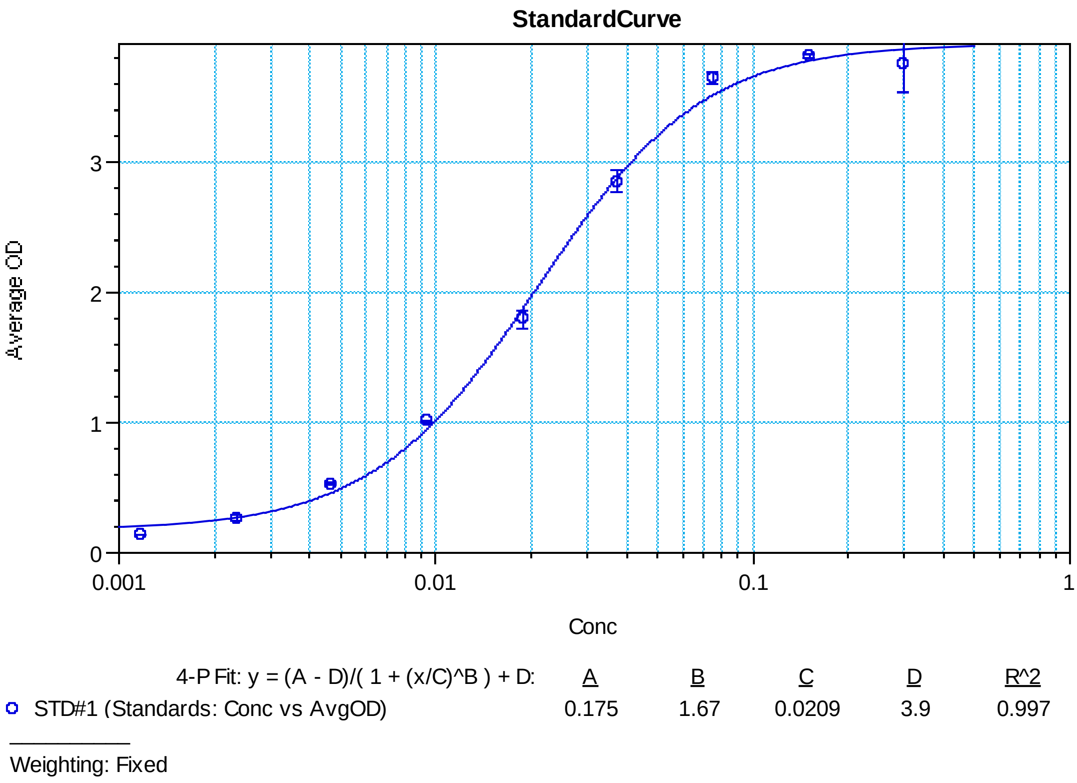

Figure S5: A representative 4-parameter logistic plot of IL-1β standard samples of 9 points showing the values of a, b, c, and d constants and the calibration equation with a good fit (R2=0.997). The data represents the mean ± SD of optical density (OD) values for duplicate standard concentrations (n=2).

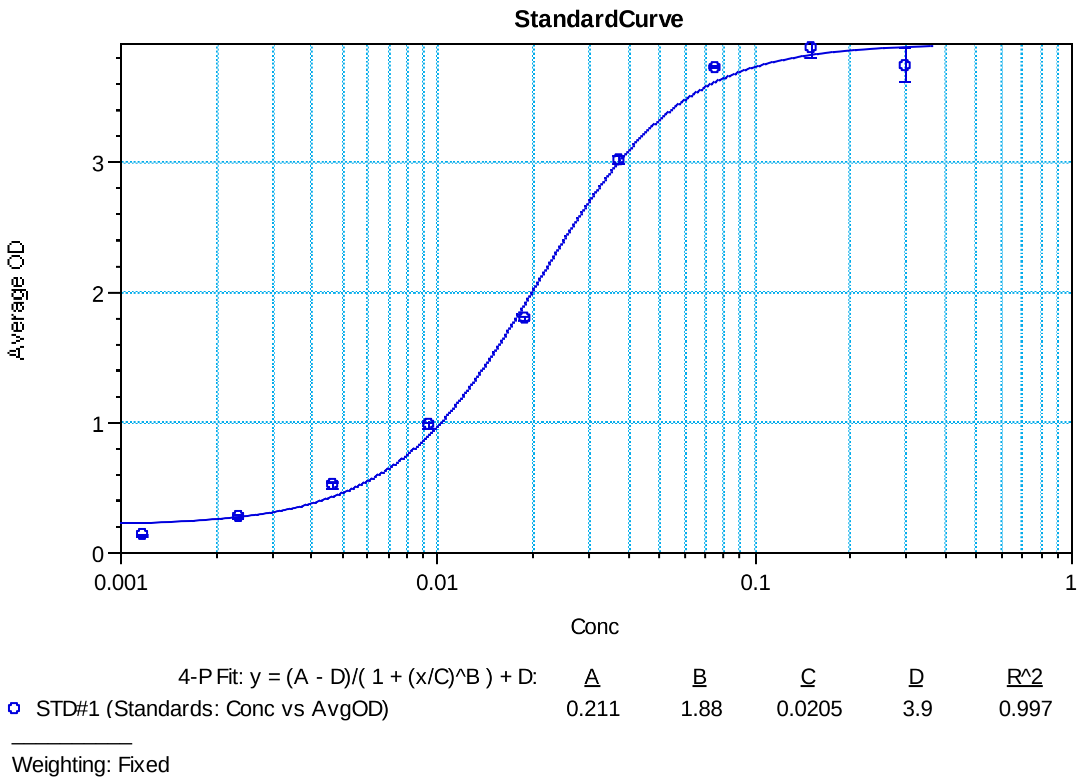

Figure S6: A representative 4-parameter logistic plot of IL-1β standard samples of 9 points showing the values of a, b, c, and d constants and the calibration equation with a good fit (R2=0.997). The data represents the mean ± SD of optical density (OD) values for duplicate standard concentrations (n=2).

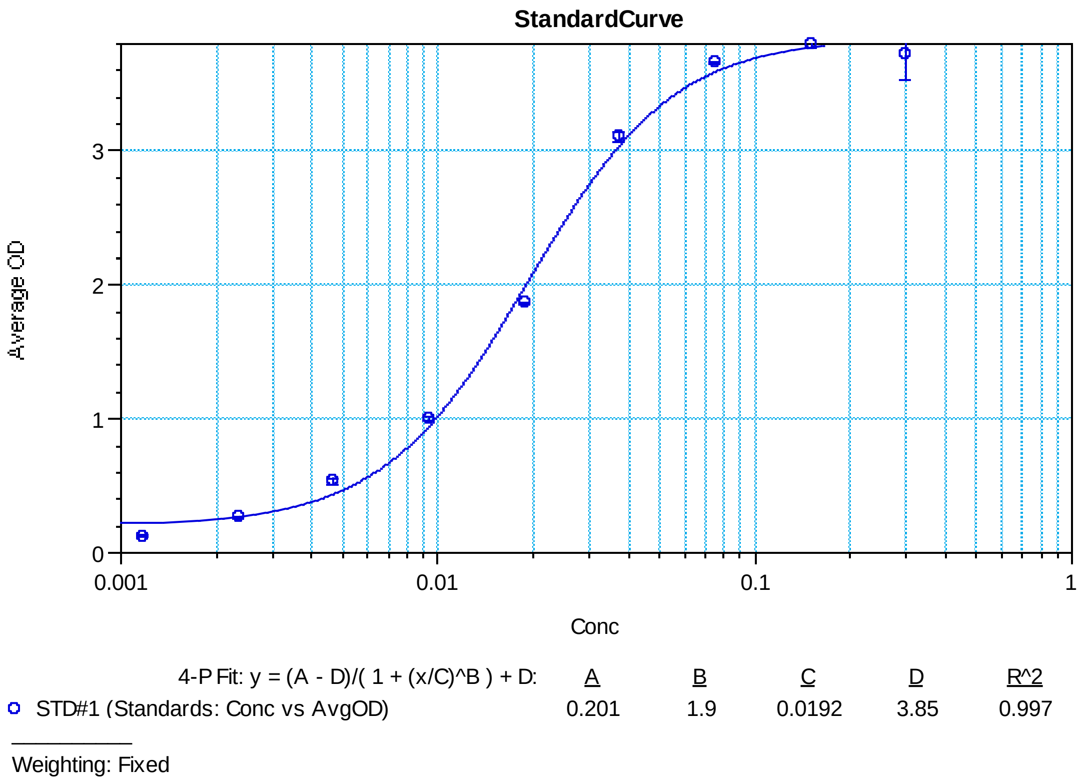

Figure S7: A representative 4-parameter logistic plot of IL-1β standard samples of 9 points showing the values of a, b, c, and d constants and the calibration equation with a good fit (R2=0.997). The data represents the mean ± SD of optical density (OD) values for duplicate standard concentrations (n=2).

IL-6 production

Table S3: Effect of propolis extracts on the production of IL-6 cytokines in the presence and absence of LPS on PMA-differentiated THP-1 cells (n=3).

	IL-6 concentration (pg/ml)									
	**Sample only**					**Sample + LPS**				
**Propolis Samples**	**n=1**	**n=2**	**n=3**	**Mean**	**RSD**	**n=1**	**n=2**	**n=3**	**Mean**	**RSD**
**P-UK1**	<2.0	<2.0	<2.0	n/a	n/a	13	11	12	12.00	8.33
**P-UK2**	<2.0	<2.0	<2.0	n/a	n/a	47	37	49	44.33	14.50
**P-UK3**	<2.0	<2.0	<2.0	n/a	n/a	46	34	49	43.00	18.46
**P-UK4**	<2.0	<2.0	<2.0	n/a	n/a	55	48	65	56.00	15.26
**P-UK5**	<2.0	<2.0	<2.0	n/a	n/a	53	45	49	49.00	8.16
**P-G**	<2.0	<2.0	<2.0	n/a	n/a	19	20	20	19.67	2.94
**P-C**	<2.0	<2.0	<2.0	n/a	n/a	26	25	20	23.67	13.58
**P-Ind1**	<2.0	<2.0	<2.0	n/a	n/a	50	39	41	43.33	13.52
**P-Ind2**	<2.0	<2.0	<2.0	n/a	n/a	1	<2.0	1	1	n/a
**Media**	<2.0	<2.0	<2.0	n/a	n/a					
**LPS**	111	113	82	102	17.01					

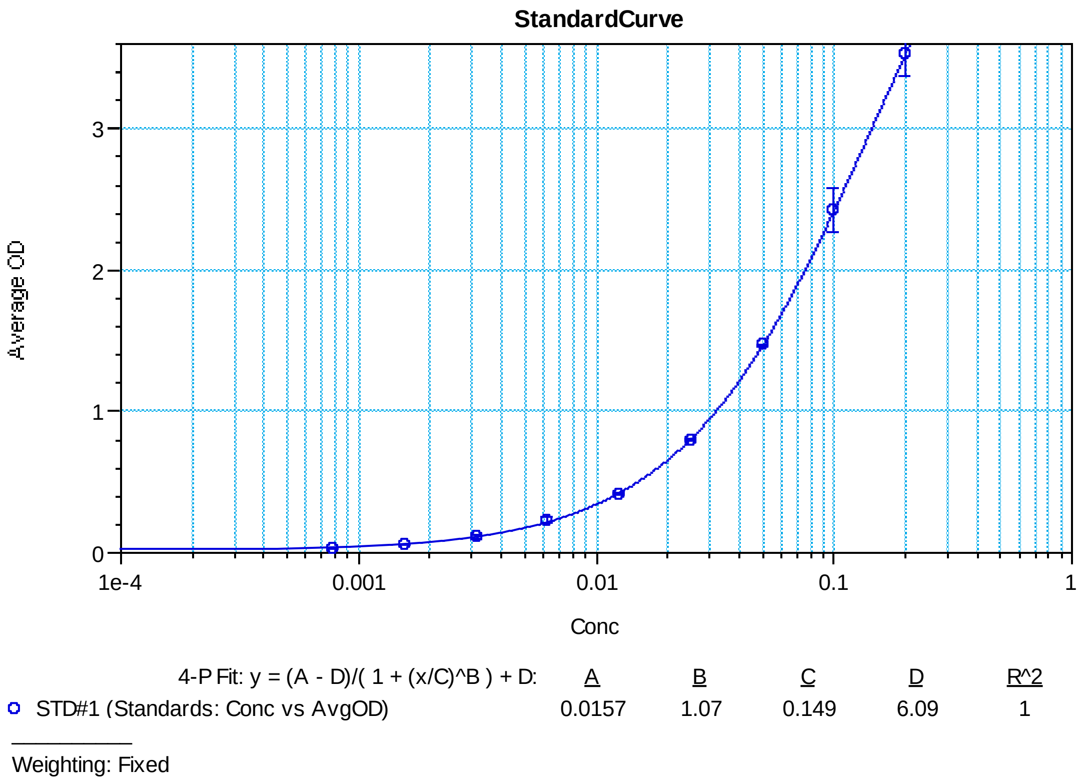

Figure S8: A representative 4-parameter logistic plot of IL-6 standard samples of 8 points showing the values of a, b, c, and d constants and the calibration equation with a perfect fit (R2=1). The data represents the mean ± SD of optical density (OD) values for duplicate standard concentrations (n=2).

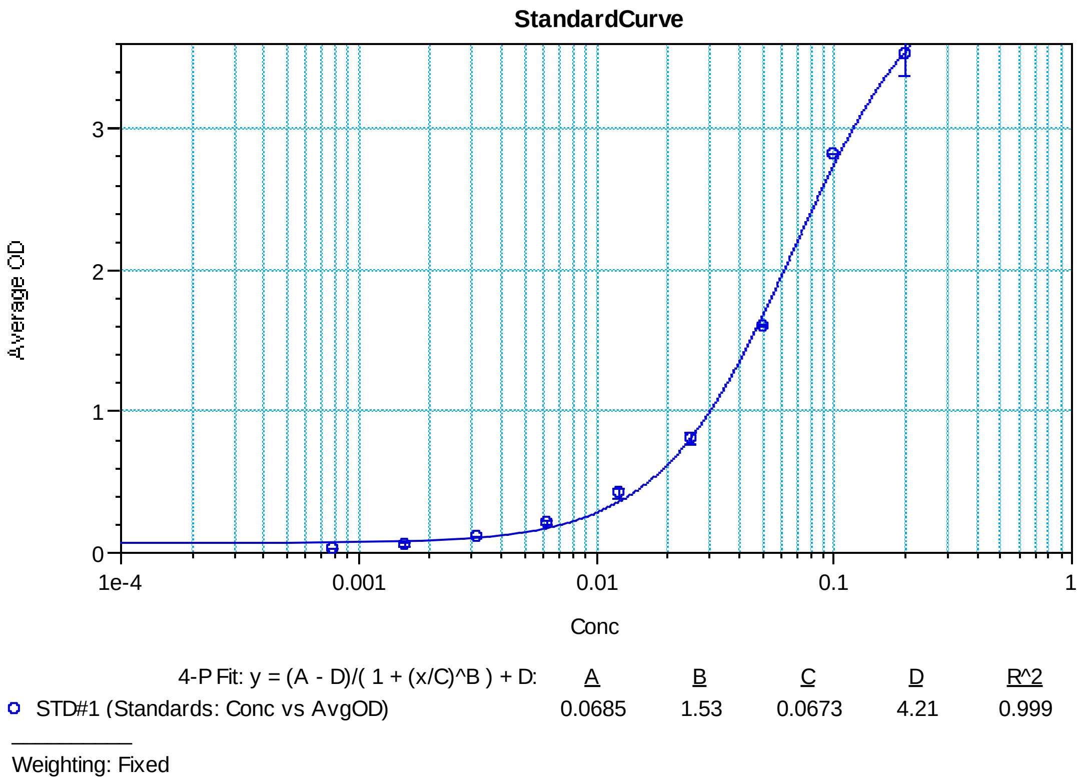

Figure S9: A representative 4-parameter logistic plot of IL-6 standard samples of 8 points showing the values of a, b, c, and d constants and the calibration equation with a perfect fit (R2=0.999). The data represents the mean ± SD of optical density (OD) values for duplicate standard concentrations (n=2)

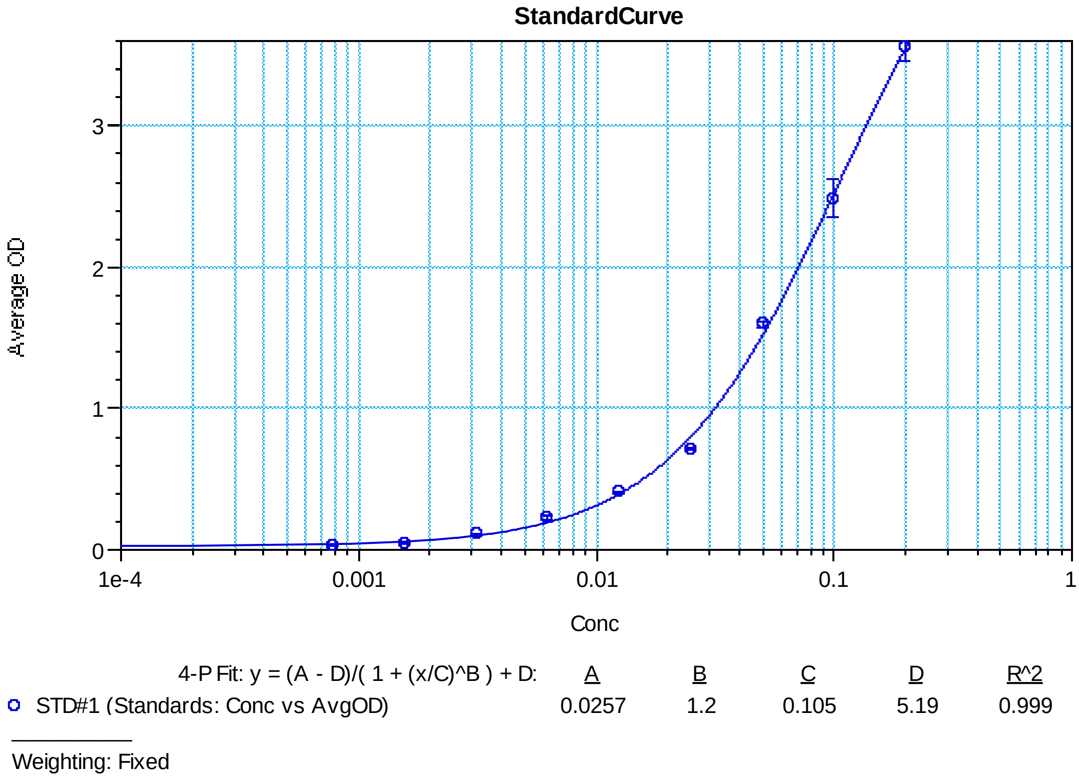

Figure S10: A representative 4-parameter logistic plot of IL-6 standard samples of 8 points showing the values of a, b, c, and d constants and the calibration equation with a perfect fit (R2=0.999). The data represents the mean ± SD of optical density (OD) values for duplicate standard concentrations (n=2).

IL-10 production

Table S4: Effect of propolis extracts on the production of IL-10 cytokines in the presence and absence of LPS on PMA-differentiated THP-1 cells (n=3).

	IL-10 concentration (pg/ml)									
	**Sample only**					**Sample + LPS**				
**Propolis Samples**	**n=1**	**n=2**	**n=3**	**Mean**	**RSD**	**n=1**	**n=2**	**n=3**	**Mean**	**RSD**
**P-UK1**	16	19	5	13.33	55.28	18	10	7	11.67	48.74
**P-UK2**	21	25	7	17.67	53.50	16	13	11	13.33	18.87
**P-UK3**	11	26	6	14.33	72.62	14	12	10	12.00	16.67
**P-UK4**	14	17	8	13.00	35.25	19	15	13	15.67	19.50
**P-UK5**	15	25	8	16.00	53.40	17	14	11	14.00	21.43
**P-G**	9	12	4	8.33	48.50	13	8	7	9.33	34.44
**P-C**	19	25	6	16.67	58.28	33	14	10	19.00	64.67
**P-Ind1**	14	18	5	12.33	53.99	31	17	11	19.67	52.19
**P-Ind2**	6	2	<2.0	4.00	70.71	<2.0	<2.0	<2.0	n/a	n/a
**Media**	15.00	14.00	10.00	13.00	20.35					
**LPS**	30.00	21.00	26.00	25.67	17.57					

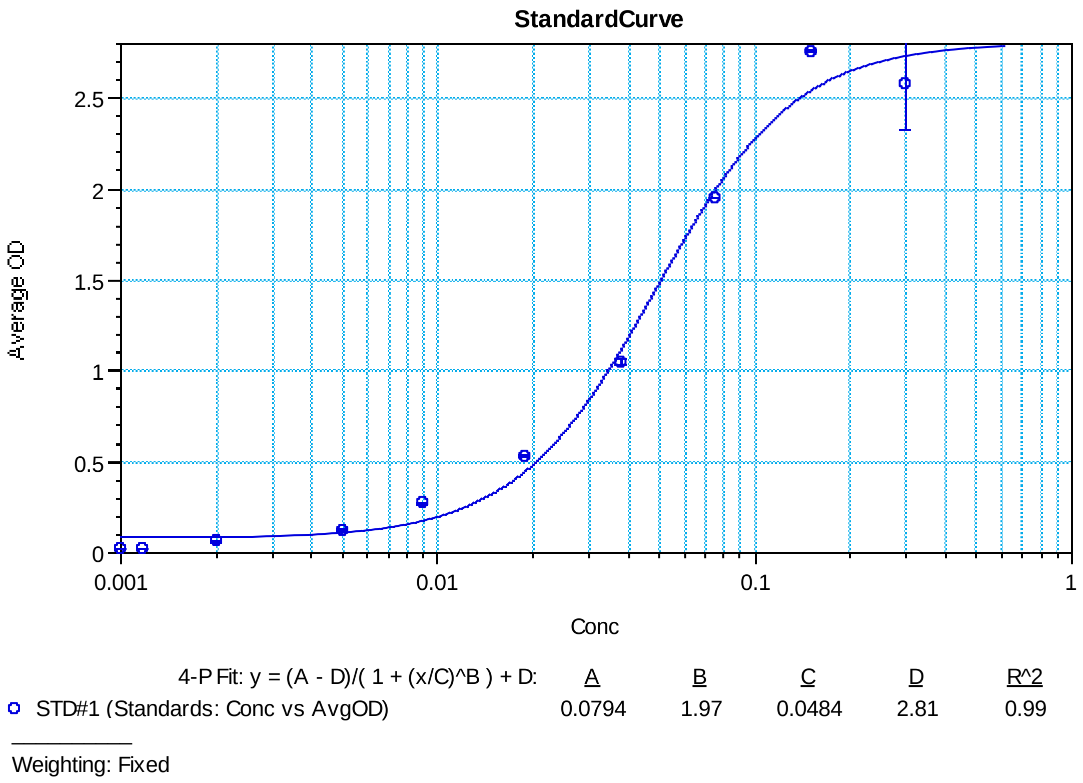

Figure S11: A representative 4-parameter logistic plot of IL-10 standard samples of 9 points showing the values of a, b, c, and d constants and the calibration equation with a good fit (R2=0.99). The data represents the mean ± SD of optical density (OD) values for duplicate standard concentrations (n=2).

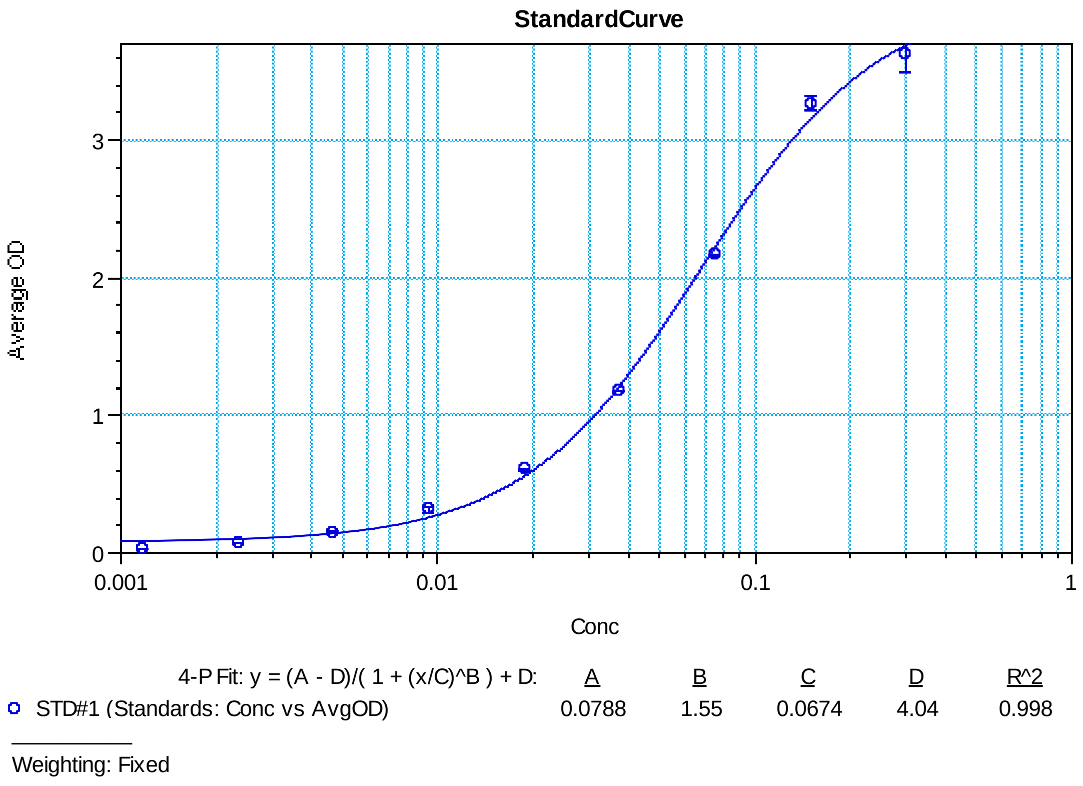

Figure S12: A representative 4-parameter logistic plot of IL-10 standard samples of 9 points showing the values of a, b, c, and d constants and the calibration equation with a good fit (R2=0.998). The data represents the mean ± SD of optical density (OD) values for duplicate standard concentrations (n=2).

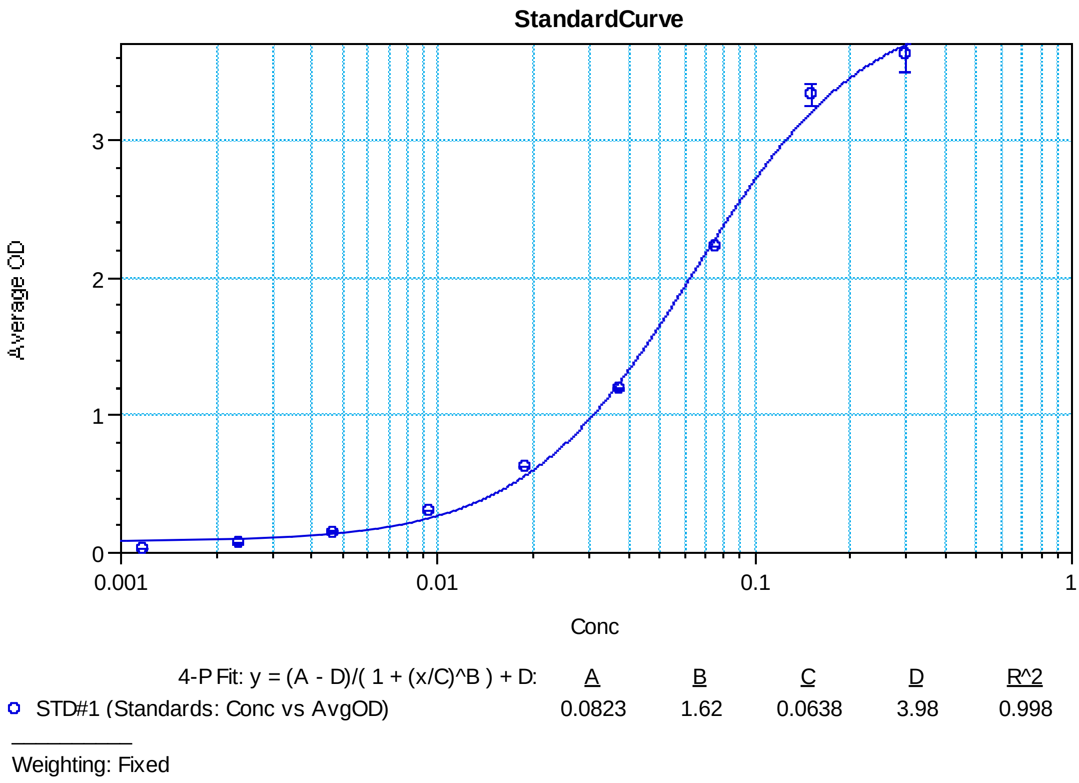

Figure S13: A representative 4-parameter logistic plot of IL-10 standard samples of 9 points showing the values of a, b, c, and d constants and the calibration equation with a good fit (R2=0.998). The data represents the mean ± SD of optical density (OD) values for duplicate standard concentrations (n=2).

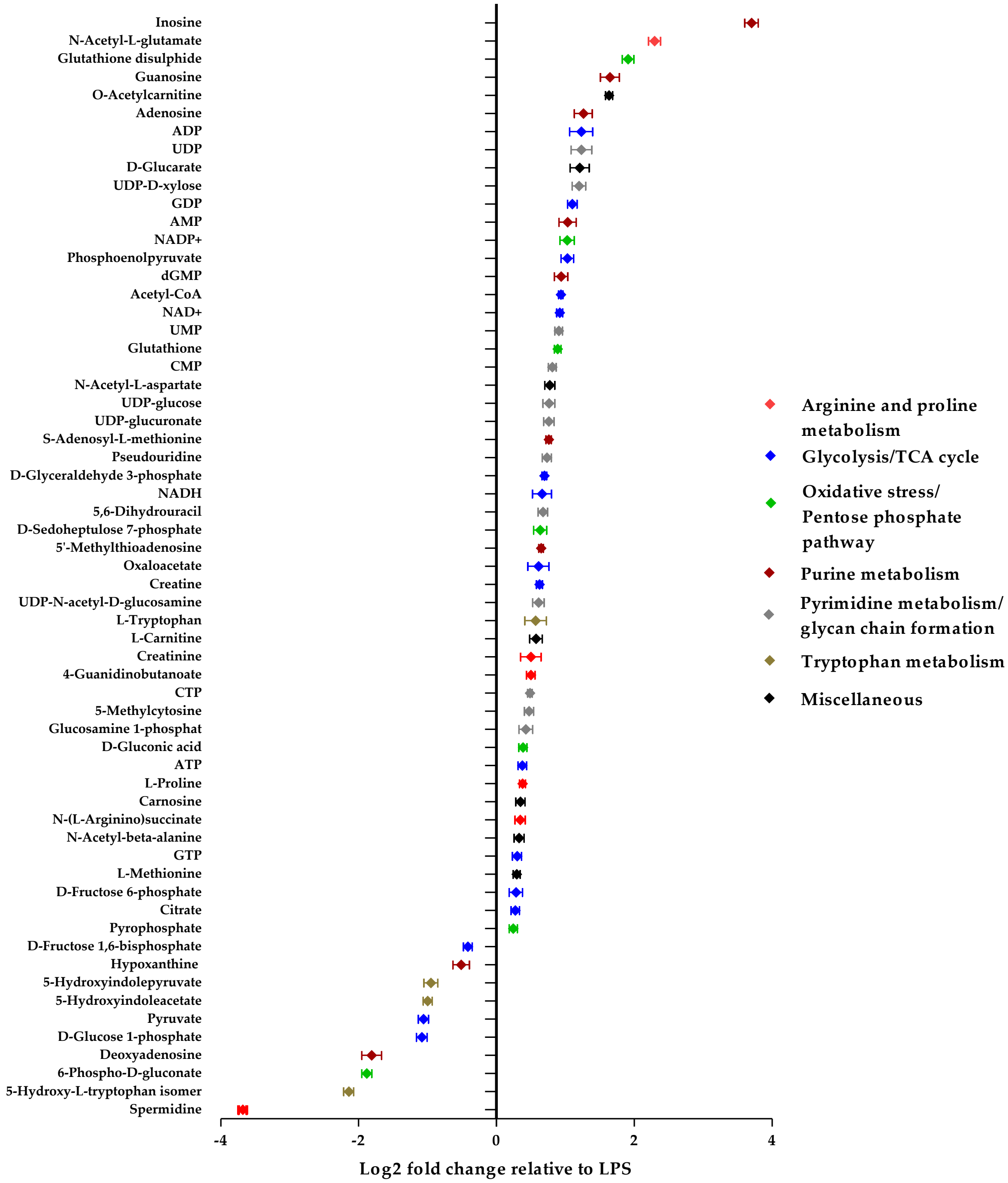

Figure S14: The log2- fold change between P-C+LPS and LPS alone in PMA-differentiated THP-1 cells. The y-axis plots individual metabolites. The x-axis plots log2 transformed relative ratio of abundance of each metabolite in the P-C and LPS combination treatment normalized to the levels of the metabolite in the positive control LPS.

Table S5: List of abbreviation used in this study.

**List of Abbreviations**	
HILIC	Hydrophilic Interaction Liquid Chromatography
RP	Reversed Phase
HPLC	High Performance Liquid Chromatography
LC-MS	liquid chromatography-mass spectrometry
UPLC-MS	Ultra-Performance Liquid Chromatograph- Mass Spectrometer
ELISAs	Enzyme-linked immunosorbent assay
SIMCA	Soft-Independent Modelling of Class Analogy
OPLS-DA	Orthogonal Partial Least Squares Discriminant Analysis
PCA	Principal Component Analysis
QC	Quality control
RT	Retention Time
PBS	Phosphate Buffered Saline
KEGG	Kyoto Encyclopedia of Genes and Genomes
TCA	Cycle Tricarboxylic Acid cycle
OXPHOS	Oxidative phosphorylation
ATP	Adenosine Triphosphate
ADP	Adenosine Diphosphate
NAD+	Nicotinamide Adenine Dinucleotide (oxidised)
NADH	Nicotinamide Adenine Dinucleotide (reduced)
NADP+	Nicotinamide Adenine Dinucleotide phosphate (oxidised)
NADPH	Nicotinamide Adenine Dinucleotide phosphate (reduced)
IMP	Inosine monophosphate
AMP	Adenosine monophosphate
CDP	Cytidine diphosphate
CTP	Cytidine Triphosphate
GTP	guanosine 5'-triphosphate
UTP	Uridine-5'-triphosphate
UDP	Uridine diphosphate
UMP	Uridine monophosphate
PMA	Phorbol 12-myristate 13-acetate
LPS	Lipopolysaccharide
PAMPs	Pathogen-associated molecular patterns
TLRs	Toll-like receptors
IDO	indole dioxygenase
PNP	purine nucleoside phosphorylase
AHR	aryl hydrocarbon receptor
ROS	Reactive oxygen species
iNOS	Nitric oxide synthase
NO	Nitric oxide
HIF-1α	Hypoxia inducible factor-1α
NF-κB	Nuclear factor kappa B

Table S6: List of catalog/serial number of instruments and reagents used in this study.

	**Catalog/serial numbers**
HPLC	5035.0016
MS	SN01059P
Reveleris® iES system	1912L00078
plate reader	MV02120
ZIC-pHILIC column	543895
TNF- α ELISA Ready-Set-Go kits	88-7346-88
IL-1β ELISA Ready-Set-Go kits	88-7261-88
IL-6 ELISA Ready-Set-Go kits	88-7066-88
IL-10 ELISA Ready-Set-Go kits	88-7106-88
RPMI 1640 media	15-040-CVR
foetal calf serum	F13-1090/500
L-glutamine solution	RNBF8011
Penicillin/Streptomycin	015M4769V

© 2019 by the authors. Submitted for possible open access publication under the terms and conditions of the Creative Commons Attribution (CC BY) license (http://creativecommons.org/licenses/by/4.0/).
